# Cortical neuromodulation by vibration-induced illusion of movement is observed in older individuals, but not in individuals with acute stroke: a cross-sectional fNIRS study

**DOI:** 10.1186/s12984-026-02129-w

**Published:** 2026-08-31

**Authors:** Brieuc Léger, Pascal Auzou, Élodie Fourdrinoy, Mathilde Sarrazin, Sylvine Celot, Céline Gay, Barbara de Dieuleveult, Clara Cohen, Canan Özsancak, Stéphane Perrey

**Affiliations:** 1 Service de Neurologie Et Unité Neurovasculaire, Centre Hospitalier Universitaire d’Orléans, 14 Avenue de L’Hôpital, 45067 Orléans Cedex 2, France; 2 EuroMov Digital Health in Motion, Univ Montpellier, IMT Mines Ales, Montpellier, France; 3 Université d’Orléans, Li2RSO, 14 Avenue de L’Hôpital, 45067 Orléans Cedex 2, France; 4 Service de Neuroradiologie, Centre Hospitalier Universitaire d’Orléans, 14 Avenue de L’Hôpital, 45067 Orléans Cedex 2, France

**Keywords:** Vibration, Illusion, Proprioception, fNIRS, Acute stroke, Aging

## Abstract

**Background:**

Together with the aging of the world population, stroke incidence is expected to increase in the coming years. Stroke is frequently associated with sensorimotor impairments of the upper limb, including proprioceptive deficits, that substantially impair motor control and quality of life. Understanding how physiological aging and acute stroke affect proprioceptive processing is therefore crucial for developing targeted rehabilitation strategies.

**Methods:**

Vibration-induced illusion of movement was administered to 28 young, 30 older, and 23 individuals with acute stroke to the distal tendon of the triceps brachii. In healthy participants, vibration was applied to both arms, whereas in participants with acute stroke, it was applied to the paretic left arm. Vibration-induced illusion of movement was delivered either with or without visual feedback of the vibrated arm. Each condition consisted of six 15-s vibrations at 80 Hz. The illusion was quantified using the Standardized Kinesthetic Illusion Procedure scale, while cerebral hemodynamic activity within frontoparietal and sensorimotor areas was assessed using fNIRS through changes in (de)oxyhemoglobin concentrations. Subsequently, activation profiles were established, and group cerebral hemodynamics and functional neural efficiency were compared.

**Results:**

Older adults exhibited more extensive bilateral activation in the frontoparietal and sensorimotor cortices compared with young adults, suggesting age-related changes in neural recruitment during proprioceptive processing. In contrast, participants with acute stroke showed no significant task-related cortical activation and displayed an overall reduction in cerebral activity compared with both healthy groups. Despite this reduced activation, neural efficiency in participants with acute stroke was comparable to that observed in older participants.

**Conclusion:**

Together, these findings suggest that preserved compensatory mechanisms may still be engaged early after stroke, and suggest potential implications of vibration-induced illusion of movement to limit age-related proprioceptive decline and to support early rehabilitation after stroke.

*Clinical Trial registration*: NCT06218563—2024-01-12.

**Graphical Abstract:**

**Figure:**
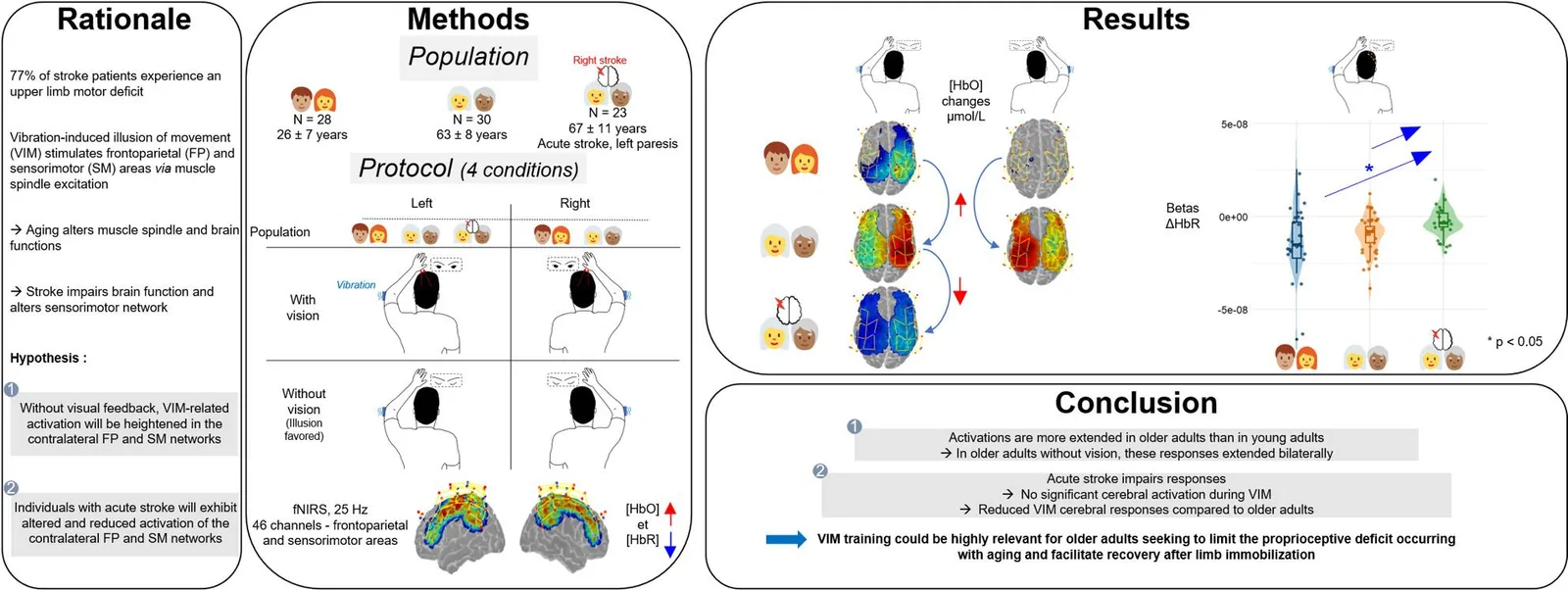

**Supplementary Information:**

The online version contains supplementary material available at https://doi.org/10.1186/s12984-026-02129-w.

## Introduction

As the global population continues to age [1], predictive models indicate that approximately one in four individuals over the age of 25 will experience a stroke during their lifetime [2]. Additionally, the incidence of ischemic strokes is projected to rise among both men and women of all ages during the current decade [3]. Furthermore, 77.4% of the affected individuals exhibit upper limb motor deficits [4], with approximately 50% retaining moderate to severe motor deficits after a period of four years [5]. In addition, proprioceptive deficits of the upper limbs have been reported in up to 63% of individuals with a stroke [6]. Together, these persistent sensorimotor impairments substantially contribute to post-stroke disability and reduced quality of life. Indeed, 67% of individuals report the non-use or disuse of their affected arm as a major problem [5].

In this context, tendinous vibration emerges as a promising proprioceptive stimulation approach with neuromodulatory effects, capable of preventing alterations of the cerebral sensorimotor network after limb immobilization [7–9] and promoting motor function recovery [10]. Indeed, tendinous vibration has been demonstrated to elicit a stimulatory effect on the intrafusal group 1a fibers of the muscle spindle [11]. Subsequently, a muscle stretch signal is transmitted to the sensorimotor and frontoparietal cortical regions, which have been identified as the mediators of both voluntary and illusory movements, the sensation of movement, and the urge to move [12–16]. This mechanism has been demonstrated to create an illusion of movement when the tendon is vibrated, at an optimal frequency of 70–80 Hz [13, 17, 18]. However, the vividness of the illusion can be mitigated when the vibrated limb is visible to the subject, as vision provides more reliable information and is deemed to dominate proprioception [19, 20].

This phenomenon is of considerable interest, as evidenced by the findings of numerous studies suggesting that the vibration-induced illusion of movement (VIM) elicits greater and more extensive activations predominantly in the contralateral sensorimotor and frontoparietal regions when compared to the effects of vibration without perceived illusion [15, 21, 22]. Furthermore, the velocity and intensity of the illusion have been found to be directly related to the degree of activation [15, 21, 23, 24]. These activations exhibit a right frontoparietal dominance, with motion sensation suggested to originate in the right inferior parietal lobule [12, 23, 25]. However, it was shown that the extent to which the visual stimulus attenuated the illusion can be directly proportional to the degree of activation observed in the posterior somatosensory associative area [13]. Moreover, VIM has the potential to improve the immediate performance of a reaching task in individuals with chronic stroke, suggesting a possible modification of brain activity [26]. A case study further indicates that a lesion in motor regions alters the illusion of movement up to three weeks post-stroke [27], whereas after six months the illusion experienced by the person with a stroke becomes comparable to that of healthy individuals. Although these findings suggest that the perception and neural processing of VIM may vary across post-stroke stages, the neural correlates of VIM during the acute stage remain unknown. Determining whether VIM-related sensorimotor and frontoparietal activations are preserved or altered in individuals with acute stroke is therefore essential to evaluate the potential integration of VIM into neuromodulation strategies aimed at promoting upper-limb motor recovery after stroke [28, 29].

To investigate the neural correlates of VIM, functional near-infrared spectroscopy (fNIRS) appears to be a suitable method in neuromodulation [30]. By emitting near-infrared light (760–850 nm), fNIRS enables indirect quantification of localized brain activity by measuring cortical hemodynamic changes in the brain in a similar fashion to functional magnetic resonance imaging (fMRI) [31, 32]. Concentration variations of oxyhemoglobin (HbO) and deoxyhemoglobin (HbR) can be determined a posteriori by utilizing the modified Beer-Lambert Law [33]. Indeed, fNIRS measures cortical brain activity with greater temporal resolution than fMRI [34], and higher spatial resolution than electroencephalography (EEG) [35]. Furthermore, the affordability, portability, and practicality of the tool serve to reinforce its status as a preferred instrument. Consequently, fNIRS facilitates the execution of experimental conditions in a more ecologically relevant environment (i.e., sitting/standing positions) and provides a superior framework for sensorimotor experiments [36].

Therefore, the objective of this study was to investigate how visual feedback modulates the neural correlates of VIM in healthy young individuals, in the context of physiological aging, and after acute stroke using fNIRS. To this end, a series of vibrations were applied to the distal tendon of the left and right triceps brachii, with and without visual feedback of the vibrated arm. Individuals with acute stroke were exposed exclusively to vibrations applied to the (left) paretic arm. We hypothesized that, compared with visual feedback, vibrations without visual feedback would induce heightened activation of the contralateral sensorimotor and frontoparietal networks, consistent with the perception of the illusion. Conversely, visual feedback of the immobile limb was expected to attenuate the illusion and reduce activation within the sensorimotor and frontoparietal networks while promoting activity in contralateral somatosensory regions, likely due to visuo-proprioceptive sensory conflict. Finally, compared with healthy individuals, we hypothesized that individuals with acute stroke would exhibit altered and reduced activation of the contralateral sensorimotor and frontoparietal networks because of impaired sensorimotor processing following stroke.

## Materials and methods

### Study characteristics

The present cross-sectional study was reported in accordance with the STROBE guidelines [37]. The behavioral data from this study have been previously analyzed in [38]. Healthy young and older adults, as well as individuals with acute stroke, were recruited over the course of two time periods: from January 2024 to April 2024, and from February 2024 to June 2025, respectively. All experiments were conducted in the Neurology and Vascular unit of the University Hospital Center of Orléans (France).

The Institutional Review Board (CPP Sud-Méditerranée 1) approved the study protocol on June 14 2023, and it was subsequently registered under the number 2023-A01062-43 (Jardé law, RIPH2). The study was prospectively registered on ClinicalTrials.gov, under the identifier NCT06218563. All participants provided free and informed written consent in accordance with the Declaration of Helsinki.

### Participants

Healthy individuals were recruited through a flyer distribution campaign and word of mouth. These individuals received financial compensation of 50€ for participating in the study. Participants with acute stroke were recruited after being admitted to the unit. Participants with acute stroke had to have experienced an ischemic or hemorrhagic stroke in the right hemisphere for the first time, and therapists had to note a left motor deficit in the upper limb. Additionally, therapists were required to confirm the absence of significant cognitive impairment and aphasia, though this requirement was not quantified. Participants were evaluated using the NIHSS [39] upon admission to the unit and within 24 h of the experiment. During this period, the following tests were used to assess motor ability and spatial neglect: the Fugl-Meyer Assessment for the Upper Extremity (FMA-UE, [40]), the Bells Test [41] and the Line Bisection Test [42].

All participants had to be 18–85 years old and were screened for right-handedness (score > 40) using the Edinburgh Handedness Inventory [43]. In addition, participants underwent the Thumb Localizing Test (see [44]). As in [44], the individual median score was utilized to compute group characteristics (see the Section “Demographic data”).

### Procedures

**Fig. 1:**
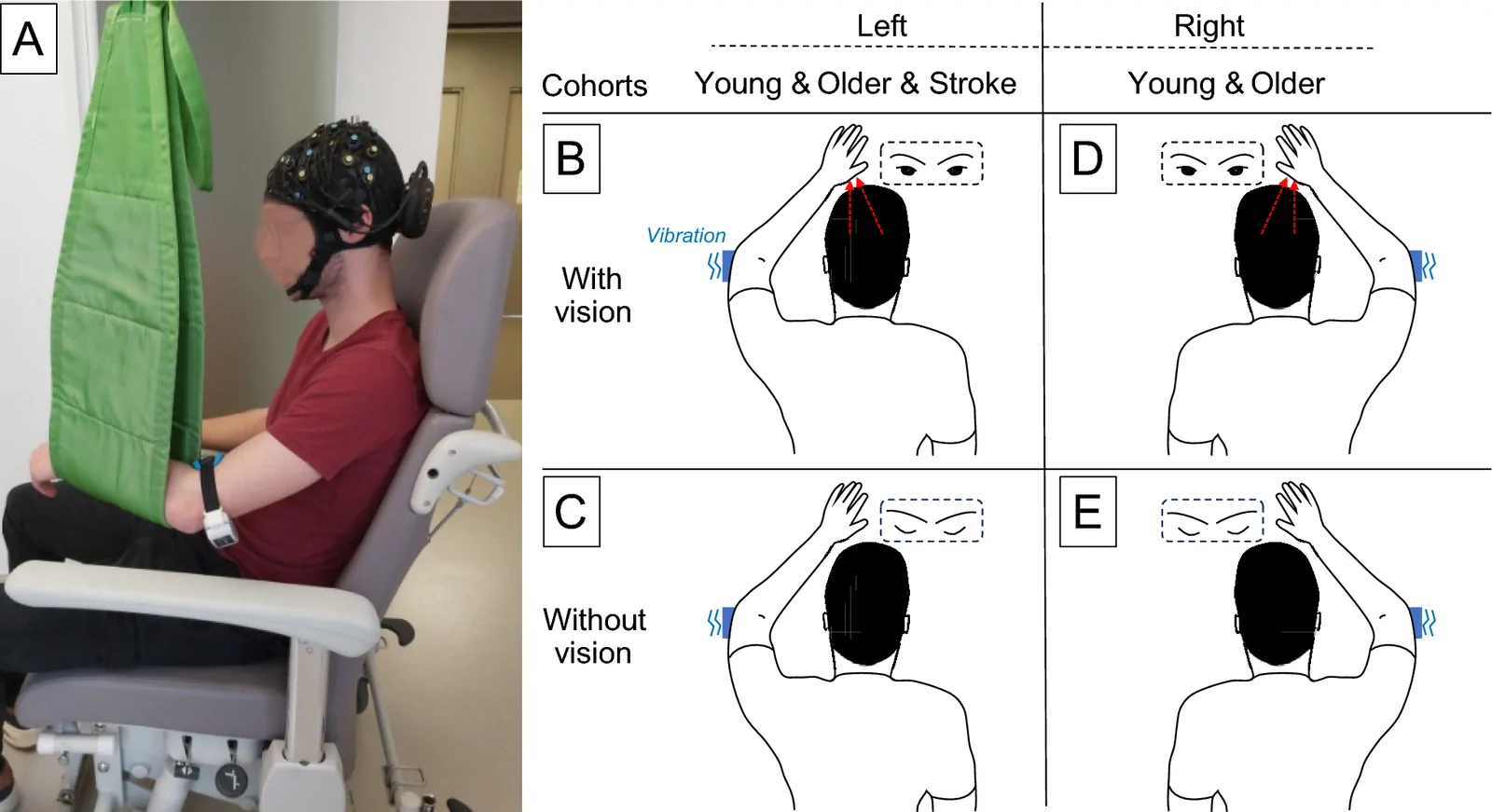
**A** Picture of the configuration in the quiet, dedicated room. A young participant is seated comfortably in the chair. The movable green strap supports the left arm; a vibrator is placed on the distal tendon of the triceps brachii muscle. Brain activity was simultaneously collected using functional near-infrared spectroscopy. **B**–**E** Schematic representation of the four modes of vibration. The vibrator is represented by the blue rectangle and the blue lines show the vibration. **B**–**C** Left arm vibration, performed in healthy young and older participants, as well as in participants with acute stroke (paretic arm). **D**–**E** Right arm vibration, performed only in healthy young and older participants. **B**–**D** Vibration with visual feedback of the vibrated arm, as indicated by open eyes and red dotted arrows pointing from the head to the hand. **C**–**E** Vibration without visual feedback of the vibrated arm, as indicated by the closed eyes

The participants were seated in a quiet, dedicated room, in a manner that ensured comfort. The subject’s left or right arm was then gently placed in a movable support strap, which exposed the distal triceps brachii tendon (see Fig. 1). This configuration was selected to prevent any disruption to the sensation of movement [45]. The Vibramoov PHYSIO vibrator device (Techno Concept, Mane, France) was placed by flexing the participant's arm and palpating the apex of the elbow to use it as a reference point. Then, the device was placed a few centimeters above this point, on the distal tendon of the triceps brachii. Participants were instructed to relax to minimize the tonic vibration response and promote the VIM [46]. Participants were also instructed to focus on the sensations experienced in the elbow joint. This approach has the potential to not only enable perception of vibrations but also facilitate discernment of illusory arm movement. Participants received a point for reporting arm flexion, the expected movement, but they were not informed of the expected direction in order to avoid introducing bias. In the visual feedback condition, participants were instructed to keep their eyes on their hand. Tendinous vibrations were applied at 80 Hz for 15 s, with an amplitude ranging from 1 to 2 mm [17, 46]. Concurrently, brain activity was measured using the fNIRS method. The experimenter positioned the fNIRS cap according to the international 10–20 EEG system, meticulously adjusting the hair to promote contact between the sources and detectors and ensuring optimal signal quality [47].

**Fig. 2:**
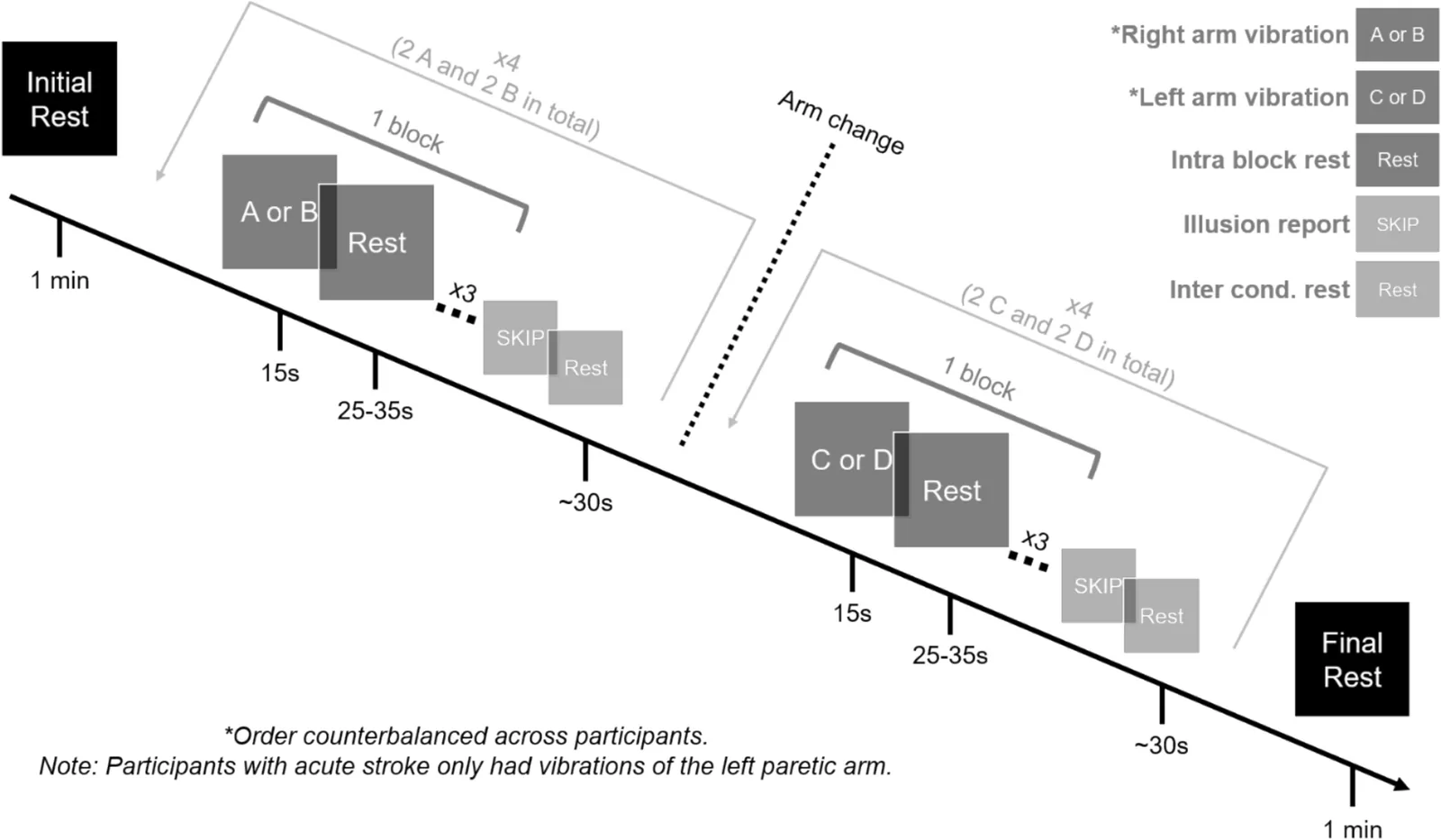
Representation of the experimental protocol. A one-minute resting period was initiated at the beginning and end of the acquisition period to ensure that the fNIRS data were not contaminated by the bandpass filter employed during signal processing. Healthy participants were administered right and left arm vibration in a counterbalanced order. Participants with acute stroke were administered vibration to their left paretic arm only. Each condition (**A**, **B**, **C** or **D**) comprised three trials of 15-s vibration, repeated twice, resulting in six vibrations in total. All vibrations were preceded by a resting period of 25–35 s. The Standardized Kinesthetic Illusion Procedure (SKIP) illusion scores were collected following each condition, after which a 30-s rest period (inter cond.) was established

In healthy participants, vibrations were applied to either the left or right arm, with or without direct visual feedback of the vibrated arm. This resulted in four distinct conditions (Fig. 1): vibration with or without vision and vibration applied to either the left or right arm. These conditions were selected based on prior evidence suggesting that visual feedback can attenuate the VIM [19, 20]. Each experimental condition (Fig. 2) consisted of six vibrations, performed in two blocks of three vibrations, for a total of 24 vibrations (2 × 3 vibrations × vision × arm). Within each group, the order of the conditions was counterbalanced. Given that participants with acute stroke may experience difficulties in maintaining concentration during prolonged tasks and may become fatigued easily, the experiments were conducted exclusively on the left paretic arm. This resulted in a total number of 12 vibrations (2 × 3 vibrations × vision). To account for the delay in the hemodynamic response to return to baseline, as well as muscle conditioning that could influence the sensation of movement and participant anticipation, a resting period varying between 25 and 35 s was selected to prevent periodic physiological noise from synchronizing with the task [46, 48, 49].

To quantify VIM responses, the Standardized Kinesthetic Illusion Procedure (SKIP) scale was employed [50]. This scale uses a four-point ordinal rating system to evaluate perceived movement at the elbow joint (0 = no illusion perceived, 1 = vague and imprecise, 2 = moderately clear and precise, 3 = perfectly clear and precise). Participants were given an additional point if they perceived movement corresponding to the expected arm flexion, yielding a possible total score of 4. When experiencing difficulty describing the direction of the perceived movement, participants were asked to reproduce it using their non-vibrated arm. SKIP scores were collected following each block of three vibrations, yielding two scores per condition. The mean of these two scores was used for subsequent analysis. The present study exclusively employed the scoring component of the SKIP procedure, which has been shown to differentiate between VIM responses in healthy individuals and in chronic stroke [50].

#### fNIRS recordings

**Fig. 3:**
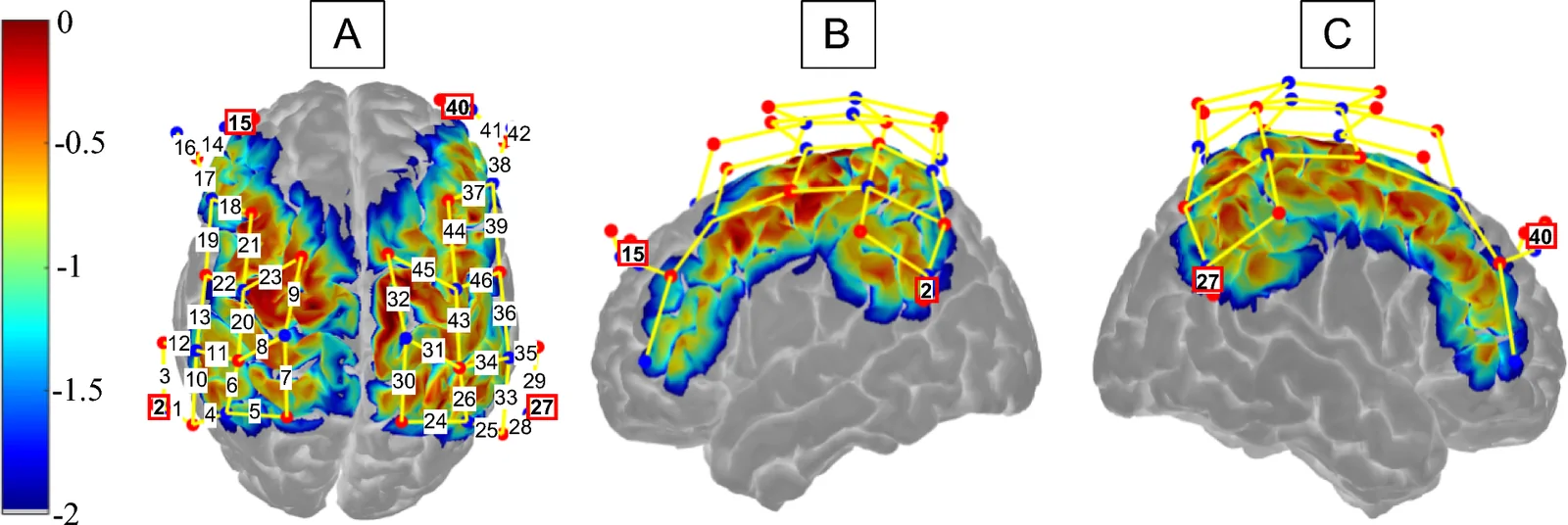
Arrangement of the 46 fNIRS channels and sensitivity maps generated with the Monte Carlo photon transport software for the depicted channel arrangement. The color bar represents the sensitivity on a logarithmic scale from 0.01 to 1 mm^−1^. **A** Dorsal view of the fNIRS sensitivity profile, showing the 46 channel numbers. Short channels are displayed in bold and are marked with red boxes (channels 2, 15, 27 and 40). Left (**B**) and right (**C**) lateral views of the fNIRS sensitivity profile

Two portable continuous-wave fNIRS systems (Brite MKII and Brite MKIII, Artinis Medical Systems, The Netherlands) were utilized to record cerebral oxy- and deoxyhemoglobin levels in the frontoparietal and sensorimotor areas. All recordings were conducted at a frequency of 25 Hz (one sample every 0.04 s) using two wavelengths of 760 and 842 nm. Each system comprised 10 LED sources and 8 light receivers, resulting in a montage with 20 LED sources and 16 light receivers. The layout was built with 46 channels (source-receiver pairs), including 42 long channels of 3 cm and 4 short channels (SC) of 1 cm (Fig. 3).

To ensure the correct positioning of the optode layout across frontoparietal and sensorimotor areas, the sources and receivers were localized in the Montreal Neurological Institute (MNI) space using a 3D digitizer (FASTRAK, Polhemus, USA). This allowed the location of each channel to be extracted onto Brodmann areas (MRIcro) using NIRS-SPM [51]. Coregistration was performed on a single representative participant prior to data acquisition. Due to slight interhemispheric inconsistencies introduced by small head movements during digitization, and despite the strictly symmetrical optode layout, the spatial coordinates of the left-hemisphere channels were mirrored to the right hemisphere to enforce geometric symmetry of the montage.

Furthermore, a sensitivity profile (Fig. 3) was generated using a Monte Carlo simulation involving 10 million photons through the tMCimg software in AtlasViewer (MatLab R2017b, The MathWorks Inc., USA) to estimate the probabilistic path of photons through the cortical surface [52].

The combined sensitivity profile and Brodmann area projections confirmed that the montage covered frontoparietal and sensorimotor areas (Fig. 3, Table 1).The Brodmann areas were grouped in six regions of interest as follows: Frontal (BAs 44, 45), dorsolateral prefrontal (dlPFC, BAs 9, 46), motor (BAs 4 and 6), primary somatosensory (S1, BAs 1, 2, 3), somatosensory association (S2, BAs 5, 7), and inferior parietal areas (BAs 39 and 40). The BAs reported in Table 1 correspond to those that exhibit the largest overlap and are all part of the same region.

**Table 1:** Spatial localization of the 46 channels obtained with NIRS-SPM from the 3D digitizer

Channels	Sources	Receivers	MNI coordinates			Brodmann areas	% Of overlap
			X	Y	Z		
1	1	1	− 70.3	− 67.62	45.53	39—Angular gyrus, part of Wernicke's area 40—Supramarginal gyrus part of Wernicke's area	0.85 0.15
2*	2	1	− 75.09	− 65.65	29.96	39—Angular gyrus, part of Wernicke's area 40—Supramarginal gyrus part of Wernicke's area	0.51 0.1
3	5	1	− 76.62	− 50.12	45.47	40—Supramarginal gyrus part of Wernicke's area	0.96
4	1	2	− 53.98	− 70.47	68.67	39—Angular gyrus, part of Wernicke's area 40—Supramarginal gyrus part of Wernicke's area	0.47 0.30
5	3	2	− 37.2	− 66.11	83.74	7—Somatosensory Association Cortex	0.98
6	4	2	− 46.45	− 56.71	82.34	7—Somatosensory Association Cortex	0.64
7	3	3	− 22.38	− 50.2	94.6	5—Somatosensory Association Cortex 7—Somatosensory Association Cortex	0.47 0.1
8	4	3	− 32.33	− 37.53	92.64	1—Primary Somatosensory Cortex 3—Primary Somatosensory Cortex	0.30 0.27
9	10	3	− 20.09	− 14.87	94.63	4—Primary Motor Cortex 6—Pre-Motor and Supplementary Motor Cortex	0.26 0.74
10	1	4	− 64.6	− 57.68	64.38	39—Angular gyrus, part of Wernicke's area 40—Supramarginal gyrus part of Wernicke's area	0.10 0.90
11	4	4	− 54.05	− 44.22	79.55	1—Primary Somatosensory Cortex 2—Primary Somatosensory Cortex 3—Primary Somatosensory Cortex	0.23 0.35 0.19
12	5	4	− 70.17	− 38.15	62.85	40—Supramarginal gyrus part of Wernicke's area	0.86
13	9	4	− 62.92	− 25.97	71.72	1—Primary Somatosensory Cortex 2—Primary Somatosensory Cortex 3—Primary Somatosensory Cortex	0.38 0.03 0.54
14	6	5	− 53.17	46.46	39.6	45—pars triangularis Broca's area	0.59
15*	7	5	− 42.89	54.47	44.78	9—Dorsolateral prefrontal cortex 46—Dorsolateral prefrontal cortex	0.37 0.60
16	6	6	− 65.3	42.44	16.2	45—pars triangularis Broca's area	1
17	6	7	− 59.3	30.43	46.87	44—pars opercularis, part of Broca's area 45—pars triangularis Broca's area	0.61 0.19
18	8	7	− 48.86	21.03	66.77	9—Dorsolateral prefrontal cortex	0.59
19	9	7	− 57.75	10.21	63.15	6—Pre-Motor and Supplementary Motor Cortex	0.82
20	4	8	− 44.02	− 30.75	86.82	4—Primary Motor Cortex	0.63
21	8	8	− 40.69	1.67	81.28	6—Pre-Motor and Supplementary Motor Cortex	1
22	9	8	− 49.44	− 11.73	79.78	4—Primary Motor Cortex 6—Pre-Motor and Supplementary Motor Cortex	0.56 0.42
23	10	8	− 30.81	− 8.48	89.45	6—Pre-Motor and Supplementary Motor Cortex	1
24	11	9	37.2	− 66.11	83.74	7—Somatosensory Association Cortex	1
25	13	9	53.98	− 70.47	68.67	39—Angular gyrus, part of Wernicke's area 40—Supramarginal gyrus part of Wernicke's area	0.35 0.29
26	14	9	46.45	− 56.71	82.34	7—Somatosensory Association Cortex	0.73
27*	12	10	75.09	− 65.65	29.96	39—Angular gyrus, part of Wernicke's area 40—Supramarginal gyrus part of Wernicke's area	0.48 0.06
28	13	10	70.3	− 67.62	45.53	39—Angular gyrus, part of Wernicke's area 40—Supramarginal gyrus part of Wernicke's area	0.82 0.17
29	15	10	76.62	− 50.12	45.47	39—Angular gyrus, part of Wernicke's area 40—Supramarginal gyrus part of Wernicke's area	0.01 0.98
30	11	11	22.38	− 50.2	94.6	5—Somatosensory Association Cortex 7—Somatosensory Association Cortex	0.51 0.07
31	14	11	32.33	− 37.53	92.64	1—Primary Somatosensory Cortex 3—Primary Somatosensory Cortex	0.27 0.26
32	19	11	20.09	− 14.87	94.63	4—Primary Motor Cortex 6—Pre-Motor and Supplementary Motor Cortex	0.30 0.70
33	13	12	64.6	− 57.68	64.38	39—Angular gyrus, part of Wernicke's area 40—Supramarginal gyrus part of Wernicke's area	0.13 0.87
34	14	12	54.05	− 44.22	79.55	1—Primary Somatosensory Cortex 2—Primary Somatosensory Cortex 3—Primary Somatosensory Cortex	0.20 0.31 0.28
35	15	12	70.17	− 38.15	62.85	40—Supramarginal gyrus part of Wernicke's area	0.77
36	20	12	62.92	− 25.97	71.72	1—Primary Somatosensory Cortex 2—Primary Somatosensory Cortex 3—Primary Somatosensory Cortex	0.36 0.03 0.54
37	16	13	48.86	21.03	66.77	9—Dorsolateral prefrontal cortex	0.65
38	18	13	59.3	30.43	46.87	44—pars opercularis, part of Broca's area 45—pars triangularis Broca's area	0.53 0.19
39	20	13	57.75	10.21	63.15	6—Pre-Motor and Supplementary Motor Cortex	0.72
40*	17	14	42.89	54.47	44.78	9—Dorsolateral prefrontal cortex 46—Dorsolateral prefrontal cortex	0.49 0.51
41	18	14	53.17	46.46	39.6	45—pars triangularis Broca's area	0.52
42	18	15	65.3	42.44	16.2	45—pars triangularis Broca's area	0.97
43	14	16	44.02	− 30.75	86.82	4—Primary Motor Cortex	0.67
44	16	16	40.69	1.67	81.28	6—Pre-Motor and Supplementary Motor Cortex	1
45	19	16	30.81	− 8.48	89.45	6—Pre-Motor and Supplementary Motor Cortex	1
46	20	16	49.44	− 11.73	79.78	4—Primary Motor Cortex 6—Pre-Motor and Supplementary Motor Cortex	0.54 0.43

Channels marked with an asterisk correspond to the four short-separation channels (two per hemisphere: one anterior and one posterior)

#### fNIRS (pre)processing

Initially, the signals underwent a preprocessing stage within the MatLab environment (MatLab R2024b, The MathWorks Inc., USA). A visual investigation was conducted, during which channels were excluded when the observed signals of light intensity (760 or 842 nm) were deemed to be of substandard quality. Moreover, the quality testing of near infrared scans (QT-NIRS) function was employed to identify poor-quality channels based on the provided quality index [53]. All QT-NIRS parameters were utilized with their recommended default values, which are as follows: bpFmin = 0.5, bpFmax = 2.5; windowSec = 5; windowOverlap = 0; quality_threshold = 0.75. The quality threshold is defined as the ratio of the number of signal windows (5 s) that meet the criteria for an acceptable scalp coupling index (> 0.8) and peak spectrum power (> 0.1) to the total number of windows. Participants were excluded from the analysis if more than 15/46 (> 33%) of their channels had been pruned previously.

The signals were then imported into the NIRSTORM toolbox [54], within the MatLab environment. The light intensity signals were converted to optical density, and movement artifacts were corrected with the Temporal Derivative Distribution Repair [55]. An infinite impulse response Butterworth bandpass filter of 3rd order (0.01–0.08 Hz) was then applied to remove the slow fluctuations (< 0.01 Hz), Mayer waves (~ 0.08–0.1 Hz), respiration (~ 0.25–0.35 Hz) and heartbeats (~ 1–1.3 Hz) from the signals [32, 48, 49, 56]. Nonetheless, given that these particular filters exhibit a transition band and that Mayer waves frequencies coincide with the hemodynamic signal of interest, along with the potential for signal contamination by task-evoked hemodynamic signals, short-channel signals were utilized to eliminate residual superficial noise through the implementation of an ordinary least squares approach (see [54, 57–59]).

Finally, the individual differential pathlength factors were computed using the formula from [33], and the optical density data were converted into HbO and HbR concentrations using the modified Beer-Lambert Law [60]. The HbO and HbR trials were corrected relative to their 5-s baseline, and averaged for further analysis. In accordance with the prevailing consensus on the reporting of fNIRS data, and in an effort to avoid bias, both the HbO and HbR chromophores were reported (see [49, 61, 62]). Given the absence of a consensus [61–63] in the existing literature regarding the preference for the utilization of HbO [64–66] or HbR [67–70] to assess neurovascular coupling (dominant in the hemodynamic response), a channel was considered as "activated" when a significant increase in HbO and/or a significant decrease in HbR was observed during the task.

### Statistics

The initial sample size (n = 30 per group) was the same as in our previous publication reporting the behavioral results of this study [38]. This was determined based on previous studies investigating the VIM, which included between 15 and 31 participants per group [50, 71, 72], as well as studies examining its neural correlates, which included 5–22 participants per group [13, 15, 21, 24, 25]. Considering that some participants could be excluded due to poor signal quality inherent to fNIRS recordings [47], this recruitment target was considered appropriate to obtain a sample size comparable to previous neuroimaging studies investigating VIM neural correlates while accounting for potential data loss.

All statistics were performed in MatLab R2024b. Parametric statistical tests were used, as these methods have been shown to be robust for sample sizes greater than five, even in cases of non-normality and unequal variances [73]. Initially, group ages were compared using a one-way ANOVA followed by a post-hoc Tukey–Kramer procedure [74] using the function *multcompare*. The Tukey–Kramer procedure was applied to all pairwise comparisons between the three groups.

Subsequently, the channel activations were determined in each condition and group using beta values derived from a generalized linear model using a simple boxcar function that did not consider the response pattern. Since it is known that there is a ~5-s delay time after a stimulus for the hemodynamic response to rise to its maximum value and to return to baseline, the analysis was conducted on the 25-s interval following the onset of the vibration. One-sample t-tests were therefore conducted on the beta values. The resulting p-values were corrected for multiple comparisons using the false discovery rate correction based on the Benjamini–Hochberg procedure [75] with the function *mafdr*, across the tested channels, separately for each group, experimental condition, and chromophore (HbO and HbR). The effect sizes were computed using the *meanEffectSize* function, which provides an unbiased estimate of Cohen’s d, also known as Hedges’ g. The Hedges’ g effect sizes were interpreted as follows: small (g = 0.20–0.50), moderate (g = 0.50–0.80) or large (g > 0.80) [76].

Beyond activation localizations, group and vision hemodynamic differences were compared separately for the left and right arms. Beta values were averaged from the contralateral regions of interest to the vibration, comprising the sensorimotor and frontoparietal areas and encompassing all channels. Subsequently, two-way mixed ANOVAs were conducted on these values, with group as the between-subject factor and vision as the within-subject factor. Effect sizes were calculated for all ANOVAs using partial eta squares (η_p_^2^), and were interpreted according to the following classification: small (0.01–0.06), moderate (0.06–0.14), or large (> 0.14). Following significant ANOVA effects, post-hoc pairwise comparisons were performed using the Tukey–Kramer procedure, which accounts for unequal group sizes and controls the family-wise error rate. For each vision condition, the Tukey–Kramer procedure was applied to all pairwise comparisons between the three groups. Pairwise effect sizes were expressed using Hedges’ g.

Consistent with the hypothesis that reduced neural activity reflects more efficient information processing for a similar behavioral outcome [77, 78], we compared group cerebral efficiency in generating the perception of the illusory movement using a functional efficiency ratio (fER) for each group. A rescaling procedure (*rescale* function) was first applied to the averaged contralateral beta values obtained during the vibration without vision condition, constraining them to the range 0–4. This was done to rescale beta values to a comparable range with the SKIP score, ensuring that neither neural nor behavioral measures disproportionately drive the ratio due to scale differences. This ratio was only investigated in the conditions without vision, as the illusory movement was expected to be maximal in the absence of visual feedback. Subsequently, the fER was derived using the following formula:

$$ {\mathrm{Functional}}\;{\mathrm{efficiency}}\;{\mathrm{ratio}} = \frac{1 + SKIP - \beta }{{1 + SKIP + \beta }} $$

The constant 1 was added to both the numerator and denominator to prevent the ratio from consistently equaling − 1 when the SKIP score was 0 (i.e., an absence of illusion). Importantly, this additive constant also ensured that variations in beta values (HbO and HbR) continued to contribute to the ratio rather than being overshadowed by the SKIP score alone. The fER group differences for the left-arm vibration were assessed using a one-way ANOVA followed by the Tukey–Kramer procedure, applied to all pairwise comparisons between the three groups. For the right arm, fER differences were examined using a Student’s t-test, as only two groups were compared (young vs. older). Finally, the fER was multiplied by − 1 for HbR to ensure consistent interpretation with a positive scaling.

## Results

### Demographic data

A total of 29 healthy young participants, 30 healthy older participants and 26 participants with acute stroke were included in the behavioral analysis [38]. However, following signal quality assessments and channel rejection, one healthy young participant (37 channels) and three participants with acute stroke (19, 44 and 46 channels) were excluded from the fNIRS analysis. This reduced the final samples to 28 healthy young, 30 healthy older participants and 23 participants with acute stroke. Participants with acute stroke had an impaired sensorimotor network; see the Supplementary materials Tables 1 and 2 for further details. All participant characteristics are presented in Table 2. Statistical comparisons of baseline characteristics between groups are provided in the Supplementary materials Table 3. Young participants had significantly more rejected channels than older participants (p < 0.001), and significantly lower DPFs than both older participants (p < 0.001) and participants with acute stroke (p < 0.001). In addition, participants with acute stroke had significantly higher Thumb Localizing Test scores than both healthy young (p < 0.001) and older (p < 0.001) participants. Finally, no group differences were found for sex (Young=Older: X^2^ = 0, p = 1; Young=Stroke: X^2^ = 0.65, p = 0.42; Older=Stroke: X^2^ = 0.35, p = 0.56) or handedness (F_(2,78)_ = 2.05, p = 0.14).

**Table 2:** Characteristics of healthy young and older participants, as well as participants with acute stroke

Characteristics	Young Mean ± SD	Older Mean ± SD	Acute stroke Mean ± SD
Sample Size (n)	28	30	23
Years of age ± SD (range)	26.29 ± 6.59 (19-39)	63.23 ± 7.88 (49-77)	67.48 ± 10.63 (48-85)
Sex (n) (female/male)	14/14	14/16	8/15
Handedness Laterality Index ± SD	86.59 ± 14.52	92.43 ± 11.44	92.57 ± 10.97
Channels rejected (n) ± SD	7.45 ± 8.78	1.01 ± 1.66	3.88 ± 4.44
Differential Pathlength Factors ± SD			
760 nm	6.19 ± 0.19	7.19 ± 0.20	7.29 ± 0.27
842 nm	5.31 ± 0.19	6.31 ± 0.20	6.42 ± 0.27
Thumb Localizing Test ± SD			
Left	0 ± 0	0 ± 0	0.52 ± 0.85
Right	0 ± 0	0 ± 0	-
Lesion			
Volume (mL) ± SD	–	–	10.56 ± 19.89
Cortical-Subcortical/Mixed (n)	–	–	22/1
Nature (n: Ischemic/Hemorrhagic)	–	–	22/1
Hemisphere (n: Left/Right)	–	–	0/23
Days since stroke ± SD (range)	–	–	6.87 ± 3.00 (1-12)
NIHSS ± SD			
Admission	–	–	7.70 ± 3.69
Experiment	–	–	5.04 ± 3.62
FMA-UE score ± SD	–	–	42.35 ± 22.30
Spatial Neglect			
Line bisection ± SD			
5 mm	–	–	1.05 ± 1.88
20 mm	–	–	9.84 ± 17.18
Bells test ± SD			
Total omissions	–	–	5.61 ± 8.06
L-R omissions	–	–	2 ± 2.98

The one-way ANOVA revealed a significant difference between the age groups (F_(2,78)_ = 198.70, p < 0.001). Tukey–Kramer tests indicated that the participants in the young age group were significantly younger than participants in the older (p < 0.001) and acute stroke (p < 0.001) groups. The latter two groups were comparable in age (p = 0.10).

### Behavioral data

In brief, the full behavioral sample [38] revealed that healthy young and older participants exhibited significantly higher SKIP total scores without than with vision in both arms (Young_left_: p < 0.001, d = 1.339; Young_right_: p < 0.001, d = 1.257; Older_left_: p < 0.001, d = 0.830; Older_right_: p < 0.001, d = 0.538). However, no significant difference was observed in participants with acute stroke (p = 0.232, d = 0.240). In addition, participants with acute stroke showed significantly lower SKIP total scores without vision than healthy of both age groups (Young: p < 0.001, d = 1.496, Older: p = 0.049, d = 0.514). Although the present fNIRS sample excluded a few participants due to signal quality issues, the behavioral patterns were consistent with those reported previously [38]. Only a slight difference was noted in the older versus stroke comparison, yielding a p-value of 0.049 in the full behavioral sample, and 0.060 in the present fNIRS sample. Nevertheless, the Cohen’s d values were nearly identical (d = 0.514 and d = 0.511), suggesting that the result is sensitive to sample size rather than representing a qualitative change.

### fNIRS data

#### Effects of vibration conditions on brain activation for each group

All p-values and Hedges’ g are reported for all channels, and for both HbO and HbR in the Supplementary materials Table 4.

**Fig. 4:**
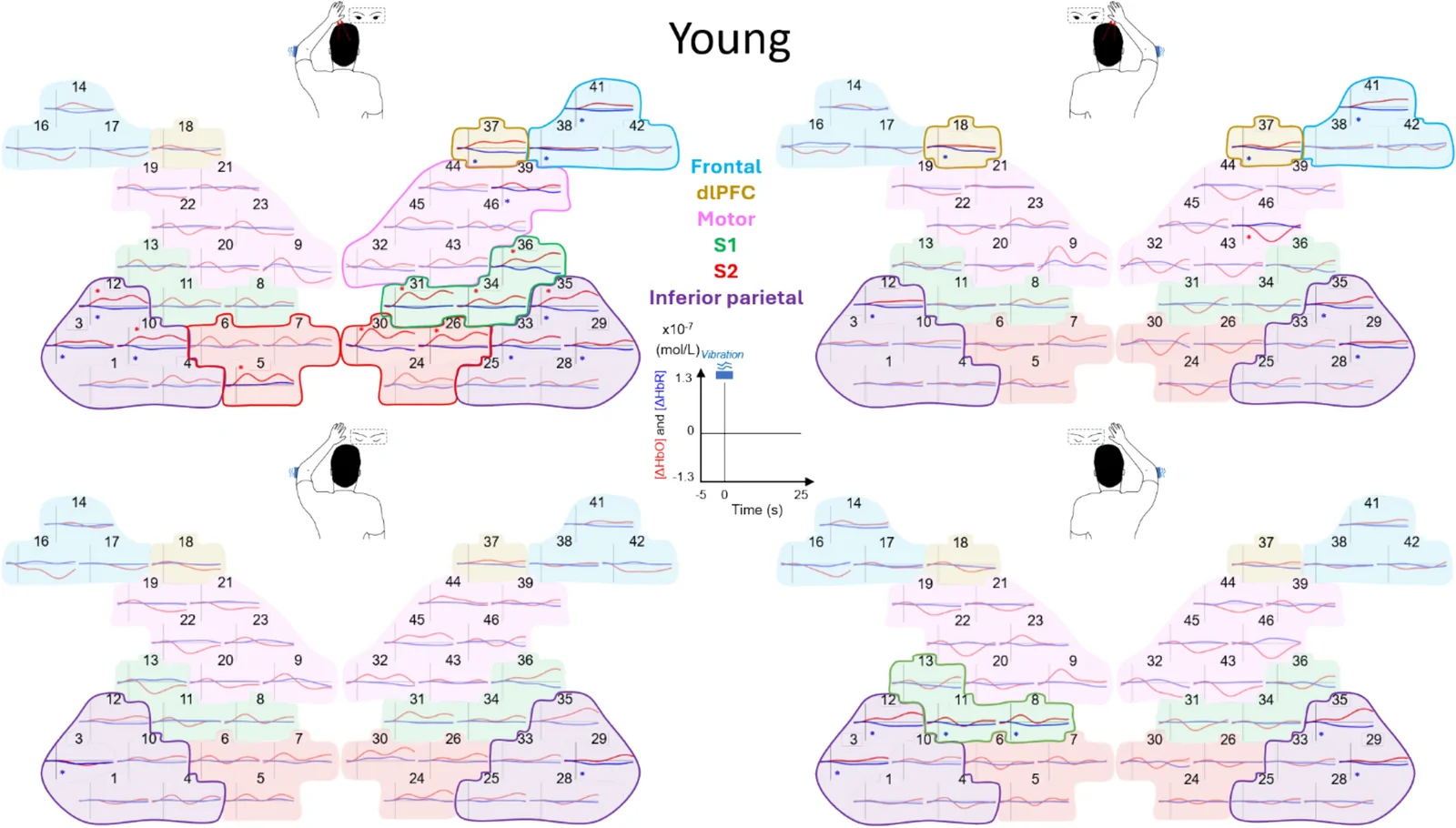
The plots show the HbO and HbR levels of healthy young participants for each channel and condition. The four different conditions are indicated by the character with left or right arm vibration and eyes open or closed. The red line shows HbO. HbR is shown as the blue line. The vertical black line indicates the start of the vibration, which begins after the five-second baseline period. The plots are displayed for 25 s after the vibration begins. Non-significant channels are semi-transparent, while channels displaying significant increases or decreases in HbO and/or HbR are opaque. Significant changes in HbO and HbR are indicated by a red or blue asterisk above (increase) or below (decrease) the channel's plot. Regions of interest are highlighted with colored backgrounds: frontal areas are blue, dorsolateral prefrontal (dlPFC) areas are dark yellow, motor areas are pink, primary somatosensory (S1) areas are green, somatosensory association (S2) areas are red, and inferior parietal areas are purple. Regions of interest containing at least one significant channel are additionally outlined with colored borders

During vibrations of the right arm with vision, significant activations were observed in the bilateral frontoparietal regions of young participants (Fig. 4). However, no frontal activations were observed in the absence of vision. Instead, activation was observed in the contralateral somatosensory regions. During the left arm vibrations with vision, significant activation of the contralateral frontoparietal and sensorimotor regions was observed, as well as activation of the contralateral somatosensory and inferior parietal regions. Interestingly, young participants exhibited solely activations in the bilateral inferior parietal regions without vision.

**Fig. 5:**
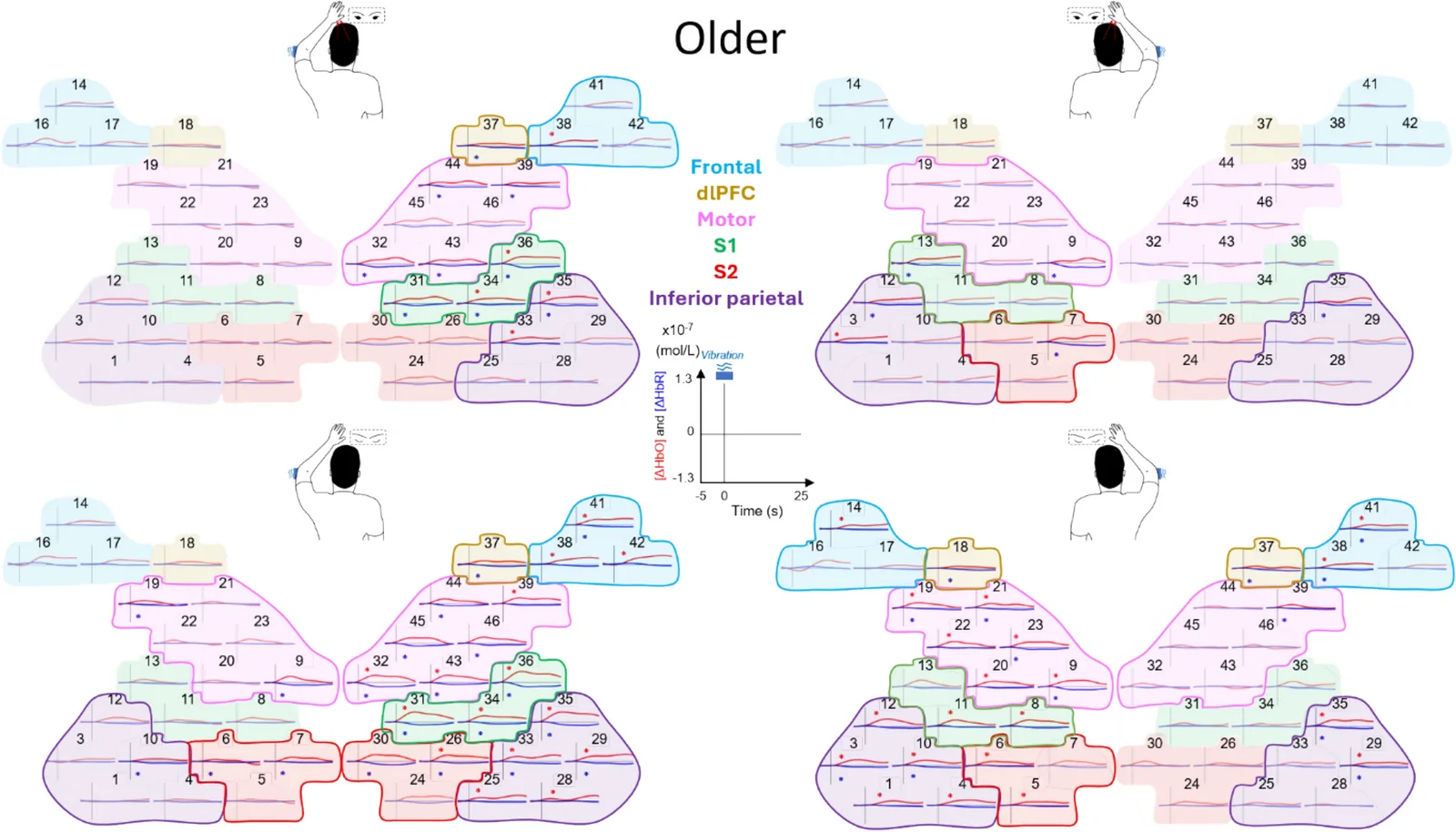
The plots show the HbO and HbR levels of healthy older participants for each channel and condition. The four different conditions are indicated by the character with left or right arm vibration and eyes open or closed. The red line shows HbO. HbR is shown as the blue line. The vertical black line indicates the start of the vibration, which begins after the five-second baseline period. The plots are displayed for 25 s after the vibration begins. Non-significant channels are semi-transparent, while channels displaying significant increases or decreases in HbO and/or HbR are opaque. Significant changes in HbO and HbR are indicated by a red or blue asterisk above (increase) or below (decrease) the channel's plot. Regions of interest are highlighted with colored backgrounds: frontal areas are blue, dorsolateral prefrontal (dlPFC) areas are dark yellow, motor areas are pink, primary somatosensory (S1) areas are green, somatosensory association (S2) areas are red, and inferior parietal areas are purple. Regions of interest containing at least one significant channel are additionally outlined with colored borders

Figure 5 shows that, in older participants, right arm vibration with vision induced significant activation in the bilateral inferior parietal regions and in the contralateral sensorimotor regions. Without vision, more extensive activation was observed in the bilateral frontoparietal and motor regions as well as activations in the contralateral somatosensory region. During left arm vibration with vision, significantly activated channels were observed in the contralateral frontoparietal and sensorimotor regions. Without vision, more extensive activation was observed in these same regions, as well as significant activations in the ipsilateral sensorimotor and inferior parietal regions.

**Fig. 6:**
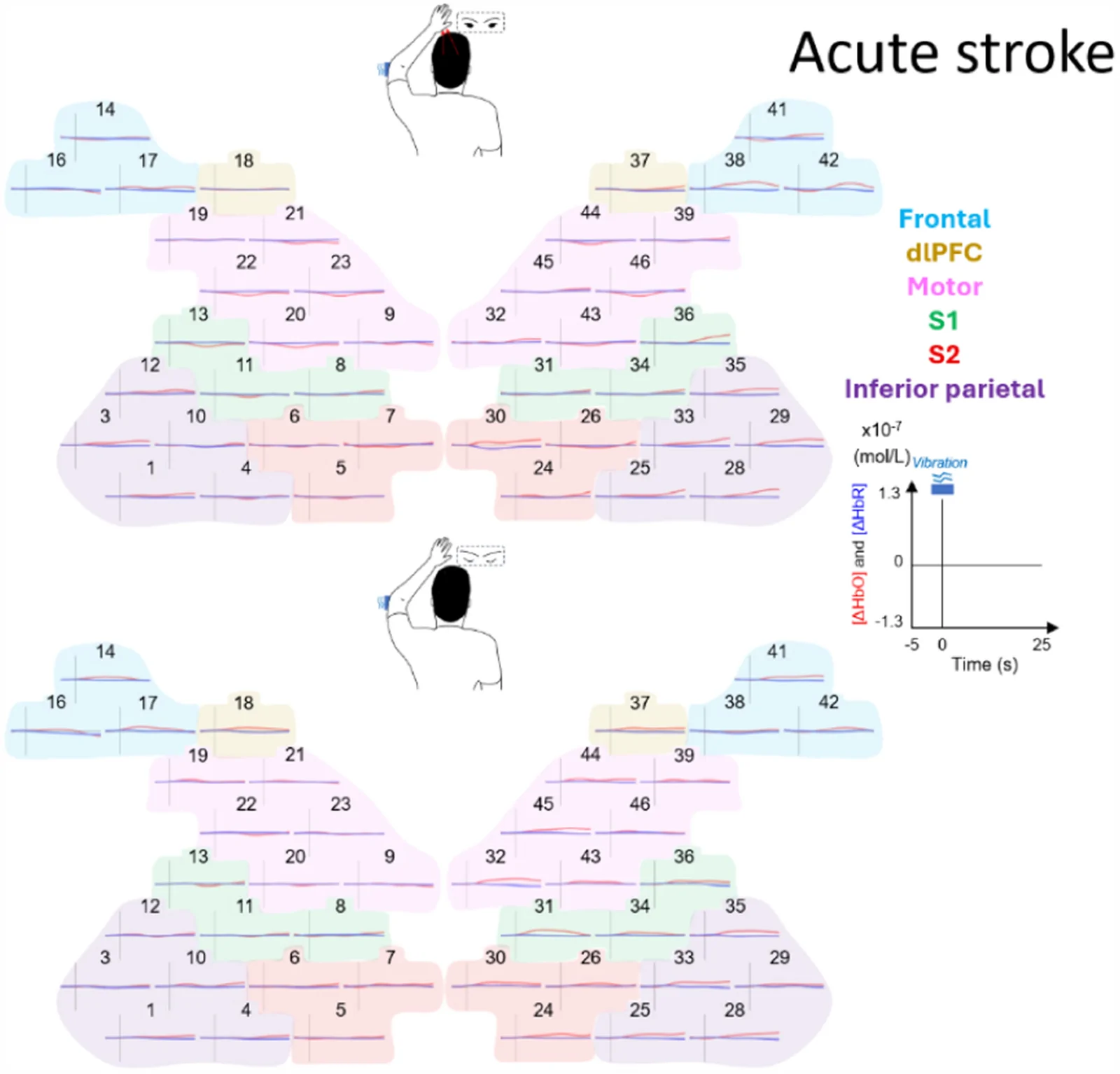
The plots show the HbO and HbR levels of participants with acute stroke for each channel and condition. The two different conditions are indicated by the character with left arm vibration and eyes open or closed. The red line shows HbO. HbR is shown as the blue line. The vertical black line indicates the start of the vibration, which begins after the five-second baseline period. The plots are displayed for 25 s after the vibration begins. HbO and HbR did not change significantly. Regions of interest are highlighted with colored backgrounds: frontal areas are blue, dorsolateral prefrontal (dlPFC) areas are dark yellow, motor areas are pink, primary somatosensory (S1) areas are green, somatosensory association (S2) areas are red, and inferior parietal areas are purple

In participants with acute stroke (Fig. 6), no significant activation was observed when the left arm was vibrated either with or without vision.

#### Group hemodynamics differences

**Fig. 7:**
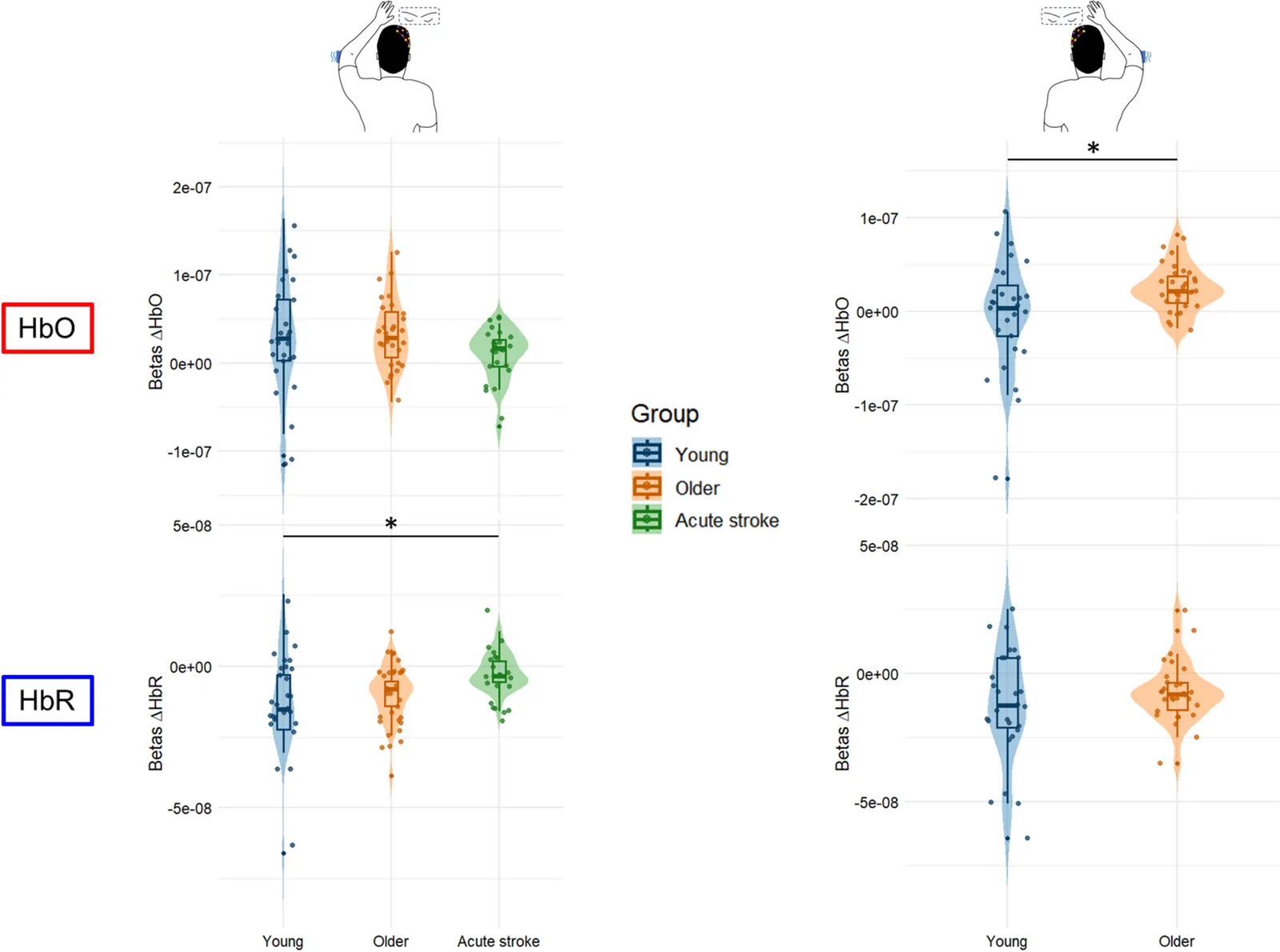
Violin plots illustrate group-related differences in contralateral hemisphere hemodynamics during left-arm (left panels) and right-arm (right panels) vibration without vision. The top row represents HbO and the bottom row HbR. Young participants are represented in blue, older participants in orange, and participants with acute stroke in green. Post-hoc Tukey–Kramer tests: * p < 0.05

During the vibration of the left arm, the two-way ANOVAs revealed that there was no significant group effect (F_(2,78)_ = 1.62, p = 0.20, η_p_^2^ = 0.04), vision effect (F_(1,78)_ = 0.00, p = 0.98, η_p_^2^ = 0.00), or interaction effect (F_(2,78)_ = 1.46, p = 0.24, η_p_^2^ = 0.04) for HbO. However, a significant group effect was identified for HbR (F_(2,78)_ = 4.74, p = 0.01, η_p_^2^ = 0.11, Fig. 7), but no significant vision effect (F_(1,78)_ = 0.50, p = 0.48, η_p_^2^ = 0.01) or interaction effect (F_(2,78)_ = 1.86, p = 0.16, η_p_^2^ = 0.05) were identified. Post-hoc Tukey–Kramer tests revealed significant differences between healthy young participants and participants with acute stroke (p < 0.05, g = 0.74, Fig. 7) but no differences were found between healthy young and older participants (p = 0.73, g = 0.17), or between healthy older participants and participants with acute stroke (p = 0.06, g = 0.96).

During the vibration of the right arm, the two-way ANOVAs revealed a significant group effect for HbO (F_(1,56)_ = 5.30, p < 0.05, η_p_^2^ = 0.09, Fig. 7). However, no significant vision effect (F_(1,56)_ = 0.004, p = 0.95, η_p_^2^ = 0.00) or interaction effect (F_(1,56)_ = 0.68, p = 0.41, η_p_^2^ = 0.01) were observed. As the group factor included only two levels, the significant main effect reflects the difference between young and older adults. The Tukey–Kramer post-hoc comparison confirmed this effect (p < 0.05) with a Hedges’ g of 0.60 (Fig. 7). Moreover, no significant group effect (F_(1,56)_ = 1.27, p = 0.26, η_p_^2^ = 0.02), vision effect (F_(1,56)_ = 0.13, p = 0.72, η_p_^2^ = 0.00), or interaction effect (F_(1,56)_ = 0.19, p = 0.67, η_p_^2^ = 0.00) was identified for HbR.

#### Functional efficiency ratio

**Fig. 8:**
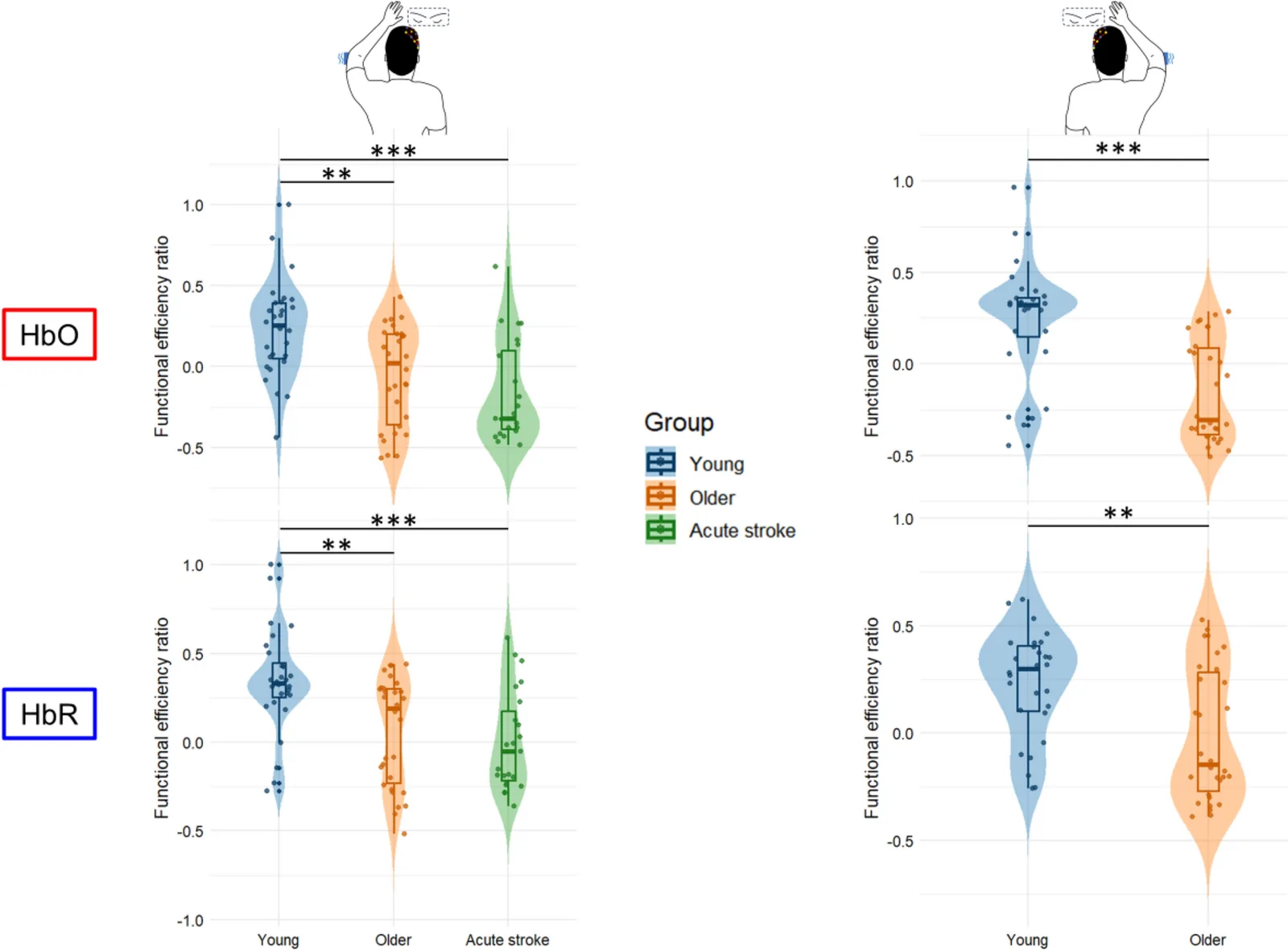
Violin plots illustrate group-related differences of the functional efficiency ratios in the contralateral hemisphere during left (left panels) and right arm (right panels) vibration without vision. The top row represents HbO and the bottom row HbR. Young participants are represented in blue, older participants in orange, and participants with acute stroke in green. Post-hoc Tukey–Kramer tests (left): ***p < 0.001, **p < 0.01; Post-hoc Student’s t-tests (right): ***p < 0.001, **p < 0.01

In the left vibration without vision condition, the one-way ANOVAs revealed significant differences for both HbO (F_(2,78)_ = 11.70, p < 0.001, η_p_^2^ = 0.23) and HbR (F_(2,78)_ = 10.30, p < 0.001, η_p_^2^ = 0.21). Post-hoc Tukey–Kramer analysis revealed that healthy older participants and participants with acute stroke had smaller fER than healthy young participants (Older: HbO: p < 0.01, g = − 0.93; HbR: p < 0.01, g = − 0.93; Acute stroke: HbO: p < 0.001, g = − 1.27; HbR: p < 0.001, g = − 1.17, Fig. 8). Furthermore, no significant differences were found in the fER between healthy older participants and participants with acute stroke (HbO: p = 0.42, g = − 0.34; HbR: p < 0.75, g = − 0.20, Fig. 8).

In the right vibration without vision condition, Student’s t-tests revealed that the fER was smaller in healthy older participants than in healthy young participants for both HbO and HbR (HbO: p < 0.001, t = − 4.95, g = − 1.28; HbR: p < 0.01, t = − 3.23, g = − 0.84, Fig. 8).

## Discussion

The present study investigated how VIM modulates cerebral activity in response to vibration of the left or right triceps brachii, with or without vision of the vibrated arm, in healthy young and older participants, as well as in the left paretic arm of participants with acute stroke. To this end, an fNIRS device was used to measure cortical hemodynamics in the frontoparietal and sensorimotor areas. Three hypotheses were examined. First, we hypothesized that the absence of visual feedback would increase activation of the contralateral sensorimotor and frontoparietal networks compared with visual feedback. This hypothesis was partially supported. In young participants, activation varied across regions and vibration sides. In older participants, however, VIM reliably activated the contralateral sensorimotor and frontoparietal areas; these responses extended bilaterally in the absence of vision. Second, we hypothesized that visual feedback would attenuate the VIM and reduce activation within the sensorimotor and frontoparietal networks while promoting activity in contralateral somatosensory regions. This hypothesis was also partially supported. At the behavioral level, visual feedback attenuated the VIM in young and older participants, as reflected by lower SKIP scores with vision. At the neural level, however, the predicted reduction in sensorimotor and frontoparietal activity and concomitant increase in contralateral somatosensory activity were not consistently observed. Finally, we hypothesized that acute stroke would be associated with altered and reduced activation of the contralateral sensorimotor and frontoparietal networks compared with healthy individuals. This hypothesis was supported, as participants with acute stroke showed no significant activation in response to VIM under any condition and exhibited lower hemodynamic activity than healthy participants, although this difference did not reach statistical significance when compared with older participants (p = 0.06, g = 0.96). Together, these findings indicate that VIM-related cortical responses are modulated by visual feedback and aging, while acute stroke is associated with a marked reduction in the cortical response to VIM.

Beyond these findings, contralateral cerebral activity in older participants in response to right-side vibration was found to be higher than in young participants. In contrast, there were no differences in the cerebral activity between young and older participants during left vibration. Furthermore, young participants demonstrated greater neural efficiency in responding to VIM than both older participants and participants with acute stroke.

### Effect of vision on cerebral activity related to the VIM

The prevailing view regarding visual feedback is that vision generally outweighs proprioceptive input, as demonstrated by studies in healthy participants using the rubber hand illusion [79], the mirror-induced visual illusion [80], and the VIM. In the latter, vision can either enhance the illusion when movement is represented by a virtual avatar [81] or attenuate it when exposed to visual feedback of the vibrated limb [19, 20]. In our study, the results were consistent with this framework, as both young and older participants exhibited stronger VIM responses in the absence of vision, whereas no such effect could be demonstrated in participants with acute stroke [38]. Given that previous studies have suggested that VIM engages a broader cerebral network than vibration without perceived illusion [15, 21, 22], we expected to observe more widespread brain activation when participants could not see their vibrated arm, particularly in healthy young and older adults.

In our sample, the results in young adults showed significant activation of the bilateral inferior parietal areas (BAs 39–40), which was consistent across conditions. These regions are classically involved in the VIM [15, 25], multisensory body representation [79, 82, 83], and have also been associated with the conscious experience of movement [12] and the sense of agency [84]. Although vision attenuated VIM responses in young participants [38], the persistence of parietal activation suggests that these areas remain a core neural substrate of the illusory movement and visual awareness. This is likely to reflect the integration of proprioceptive afferences and the predicted sensory consequences of movement.

Unexpectedly, (pre)frontal areas showed significant activation only in the presence of vision, with a right-hemispheric dominance. This result contrasts with the classical view that frontal areas constitute a core component of VIM-related networks, particularly under conditions of maximal illusion without vision. However, rather than reflecting reduced frontal area involvement in the illusory experience itself, this pattern may indicate a shift towards higher-order cognitive processes when visual feedback is available. Indeed, (pre)frontal areas have been associated with sensory conflict monitoring [82, 85]. More specifically, the dlPFC has been shown to be involved when participants perform an incongruent task involving a mismatch between their movement and the visual feedback they receive [82]. When vision is present, participants experience an explicit visuo-proprioceptive mismatch (seeing a static arm while feeling movement), which may require additional neural resources to monitor, evaluate, and resolve this sensory conflict. Furthermore, [82] reported a right dlPFC dominance during this mismatch, observing that during a visuo-proprioceptive mismatch involving passive hand movements closely resembling our experimental condition, these activations extended to the ventral frontal areas (BAs 44–45).

Furthermore, as the participants were instructed to focus explicitly on the sensation arising from their elbow joint, the observed frontal activation may reflect an enhanced awareness of bodily signals rather than the illusory movement per se. This suggests that VIM under visual feedback may involve not only bottom-up sensorimotor integration but also top-down monitoring of bodily states [82, 86]. In this context, vision may not only attenuate the illusion but also modify its neural underpinnings. This shifts the experience from a primarily parietal-driven perceptual phenomenon towards a more cognitively controlled bodily awareness process. Therefore, further research is needed to investigate and disentangle the complex cerebral network involved in the attenuation of the VIM by vision.

Furthermore, the sensorimotor areas responded differently to right- and left-arm vibration, the somatosensory areas being activated only with vision of the vibrated left arm. This finding suggests a complex interaction within the visuo-proprio-lateralized network. However, previous studies have suggested that visual guidance is more important for the right arm, whereas proprioceptive feedback is more important for the left arm [87–89]. This could explain why, during left vibration, a more proprioceptively driven cortical representation may elicit somatosensory activations, whereas such activations may be less necessary with vision of the vibrated right arm due to the dominance of visually guided control.

Conversely, older adults exhibited more widespread activation in the frontoparietal and sensorimotor areas when they were not able to see their vibrating arm than when they could see it. With vision, activations were predominantly confined to the contralateral hemisphere; without vision, however, they extended to a large part of the ipsilateral frontoparietal and sensorimotor networks, as well as recruiting more channels in the contralateral hemisphere. This phenomenon was observable during vibration of both left and right arms. These findings align with previous studies demonstrating that the frontoparietal and sensorimotor networks are more active during a clear VIM than during vibration without an illusion or an attenuated one [15, 16, 21, 22]. Rather than supporting the emergence of the illusion from a single cortical region, these results suggest that VIM in older adults probably relies on a complex interplay between frontoparietal and sensorimotor areas. It has been suggested that an increase in sensorimotor and frontoparietal networks connectivity occurs in older individuals to compensate for reduced VIM responses [24]. This could also reflect cerebral compensation mechanisms occurring with aging, whereby older adults require greater neuronal resources for a stimulus of the same intensity to achieve equivalent or lower performance than younger adults [90, 91].

Interestingly, in participants with acute stroke, the results could not demonstrate any effect of the vibration with or without vision of the vibrated arm on cerebral hemodynamics. As SKIP scores did not differ significantly with and without vision in this group, likely due to the alteration of the sensorimotor system [38], this may reflect a lack of proprioceptive stimulation for participants with acute stroke. Another factor that may have contributed is post-stroke spasticity, which was not quantified in the present study. In fact, spasticity occurs in approximately 25% of individuals early after stroke [92] and is associated with increased muscle tone and exaggerated stretch reflexes. In such cases, tendinous vibration may elicit involuntary muscle contractions, which could interfere with the perception of the VIM. Previous studies have shown that voluntary muscle contraction can attenuate the illusion [46, 93], suggesting that post-stroke spasticity may represent an additional factor influencing the perception of the VIM after stroke. Furthermore, considering the complex cerebral reorganization occurring in the acute phase of stroke [94], the absence of measured neural responses could indicate a general perturbation in cerebral hemodynamics [95]. Thus, the response may be more diffuse, and the present sample size (n = 23) may have limited sensitivity to detect small effects. To further explore this issue, we conducted a complementary post hoc analysis by averaging effect sizes across contralateral channels and estimated statistical power. As the mean HbR effect size was negligible (g = − 0.070), power estimation was performed only for HbO (g = 0.252). For this effect size, the estimated statistical power was low (0.212), suggesting that small effects may not have been reliably detected with the current sample. Power calculations further indicate that a substantially larger sample would be required to achieve conventional power levels (126 participants with acute stroke for 0.8 power, and 80 participants with acute stroke for 0.6 power). Together, these results suggest that the absence of significant findings may reflect the small effect size and diffuse nature of the response rather than a lack of physiological relevance. Furthermore, the clinical relevance of these neural differences remains unclear, as changes in cortical activation cannot be directly interpreted as functional improvements. Further longitudinal studies combining VIM neurophysiological measures with clinical outcomes are required to determine whether these neural responses may be associated with enhanced functional recovery. Therefore, future studies should consider having a larger sample size for participants with acute stroke. Furthermore, as the absence of measured significant cerebral activity in participants with acute stroke may be due to the absence of enhanced VIM, a protocol incorporating VIM associated with electrical stimulation [96–99] and/or virtual reality [81, 100, 101] to promote VIM responses could provide further insight into the neural correlates of VIM within this pathological condition.

### How does aging influence the neural correlates of VIM?

With aging, the brain is thought to adopt compensation strategies to achieve performance comparable to that of younger adults (CRUNCH model, [90, 91]). Three types of compensation have been suggested: upregulation, selection and reorganization [90].

In our study, older adults exhibited more extended activations than young adults during both left and right vibrations without vision. Furthermore, they had lower VIM responses [38] than young adults during both left and right vibrations without vision. Older adults also demonstrated stronger contralateral cerebral activity than young adults in response to right vibration without vision. These phenomena may therefore be attributable to compensation through upregulation and reorganization associated with the hemispheric asymmetry reduction occurring with aging (HAROLD model, [102]).

However, during left vibration without vision, older and young adults demonstrated comparable contralateral cerebral activity. Although there were no significant differences in left and right VIM responses between young and older adults [38], this could be explained by the left arm’s preference for proprioceptive feedback [87–89]. This preference may have required more neural resources in both groups, mitigating discrepancies. Furthermore, a previous study reported faster VIM responses for the right arm of young right-handers, though no differences were observed in older individuals [103]. This suggests that young right-handers may engage fewer contralateral neural resources with right arm vibration than left arm vibration to achieve equal performances with both arms.

Interestingly, our results showed that older participants had lower fER than young participants. This supports the idea that older adults may possess compensatory mechanisms involving upregulation to maintain their proprioceptive accuracy and keep their performance as high as possible, even though their VIM responses were still lower than those of young participants. These results align with previous studies on aging demonstrating a peripheral proprioceptive deficit through muscle spindle structural and functional alterations [104, 105], and a central proprioceptive deficit [24, 106] likely related to cortical grey matter reduction [107]. It is also worth noting that our task was passive; participants were only asked to focus on the sensation at their elbow joint. There was no expectation of maximal performance. Nevertheless, this was sufficient to highlight a reduced neural efficiency of the proprioceptive system with aging.

Finally, physical activity could prevent the aforementioned proprioceptive decline at both peripheral and central levels. This is characterized at the peripheral level by metabolic and structural adaptations of the muscle spindle [108, 109], as well as an enhanced dynamic sensitivity of muscle spindle afferents following proprioceptive training [110]. Furthermore, this is characterized at the central level through the modulation of gene expression implied in neural function [111], increased connectivity in frontal-related networks [112] and it may attenuate age-related decline in the hippocampus, temporal and frontal regions [113]. In the end, these peripheral and central adaptations could limit the proprioceptive acuity deficit occurring with aging [114–116]. Therefore, investigations of the effect of physical activity on VIM responses and neural efficiency with aging seem highly relevant.

### How does acute stroke alter neural responses to the vibration?

The included stroke participants were in the acute phase (< 14 days) and had a right (sub)cortical lesion (except for one individual, see Table 2) impairing their sensorimotor network (Supplementary materials Tables 1 and 2). Although the cerebral reorganization that occurs during the acute phase of stroke remains unclear [94], it has been documented that general cerebral hemodynamic perturbation can occur [95]. Therefore, we expected to observe decreased cerebral responses to our experimental conditions in participants with acute stroke compared with healthy young and older participants.

Indeed, our results showed that participants with acute stroke had lower neural responses to the VIM than healthy young and older participants. While this result was significant when compared with young participants, it was not significant when compared with older participants (p = 0.06, g = 0.96). Nevertheless, Hedges’ g indicated a strong effect of stroke, and this non-significant result may be due to a lack of statistical power related to the acute stroke group’s medium sample size (n = 23). In any case, it appears that acute stroke was associated with reduced contralateral neural responses to vibration. Thus, we hypothesize that acute stroke may have induced compensation by selection [90] and another cerebral network may have been recruited in response to the vibration.

Furthermore, although their fER was reduced compared with young participants, it was comparable to that of older participants. This result is noteworthy, as we expected participants with acute stroke to have decreased VIM and cerebral responses. It suggests that, despite having fewer neural resources, participants with acute stroke may preserve an acceptable level of proprioceptive acuity, which may have implications for early sensorimotor readaptation through exercise during the acute phase of stroke. Consistent with this hypothesis, a meta-analysis of Chinese persons with a stroke revealed that rehabilitation within two weeks after stroke improves motor recovery, as demonstrated by enhanced NIHSS, (modified) Barthel Index and FMA scores, compared to rehabilitation two weeks after stroke [117]. However, the authors could not determine the effects of rehabilitation at a very early stage (i.e., within 48 h) after stroke on motor recovery. Additionally, a study in hemiplegic individuals with subacute stroke revealed significant improvements in hand motor performance following a two-week VIM training [10], which persisted one month post-training. However, these authors could not demonstrate significant improvements in EEG cerebral responses at the C3 and C4 loci. More recently, Shurupova et al. (2026) investigated the effects of a three-week intervention including 12 sessions of tendinous vibration (the first two sessions also included muscle vibration) in individuals with subacute stroke (1–6 months), combined with conventional therapy, and compared with conventional therapy combined with robotic mechanotherapy [118]. The authors reported greater improvements in upper limb motor function following the vibration-based intervention. Together, these findings highlight the need for further research to better characterize how tendinous vibration training influences proprioceptive processing and its relationship with motor and cerebral recovery in individuals with acute stroke, especially over long-term outcomes.

### Methodological considerations

To minimize neural responses during the baseline period, we randomly assigned a resting period ranging from 25 to 35 s. However, to maximize the contrast-to-noise ratio, we could have shortened the resting period and task duration to allow for more trials [68]. The hemodynamic response typically peaks approximately five seconds after task onset and takes another five seconds to return to baseline [119, 120]. Therefore, a 10-s rest period between trials may be sufficient to minimize baseline noise. However, choosing appropriate resting and task periods is challenging because of the few seconds needed to perceive the illusion of movement and the short period after the end of vibration when participants may feel their arm returning [46]. Thus, we believe that a vibration duration of 10 s, interposed by resting periods ranging from 12 to 22 s, would be appropriate, as it would enable participants to perform ten trials instead of six.

Furthermore, young participants had significantly lower DPFs than both older participants and participants with acute stroke, as well as significantly more rejected channels than older participants. The observed differences in DPFs are unlikely to substantially affect the interpretation of the results, as DPFs vary with age (see [33]). Regarding the rejected channels, only 7 out of 46 channels on average were rejected in the young group, and several channels contributed to each of the six ROIs, suggesting that this difference is unlikely to have substantially affected the overall activation profiles. Additionally, EEG measurements would provide more precise insights into cerebral responses to VIM by investigating concurrent sensorimotor µ-band activity. This is particularly important in individuals with acute stroke, as the absence of an effect may be due to hemodynamic perturbations and to inter-individual variability in lesion volume and stroke severity.

Moreover, although the fER was constructed to minimize scale differences between neural (beta values) and behavioral (SKIP score) measures, the distribution of SKIP scores may still have influenced the ratio in some cases. In particular, the larger number of zero SKIP scores observed in older participants and participants with acute stroke may have reduced the relative contribution of beta values to the metric. Furthermore, participants with acute stroke had higher Thumb Localizing Test scores than healthy participants, which may reflect their neurological impairment and could represent an additional factor contributing to the observed differences in fNIRS activity. Finally, it is important to note that cognitive impairments and aphasia were not quantified in the stroke population, as these factors may have influenced the subjective reporting of the illusion of movement.

## Conclusion

In conclusion, our study revealed that VIM is associated with bilateral activation of sensorimotor and frontoparietal areas in older adults. However, acute stroke appears to alter these cerebral responses, as reflected by the absence of significant activations and reduced VIM-related cerebral responses in participants with acute stroke compared with both healthy young and older participants. Despite this reduced activation, neural efficiency in participants with acute stroke was comparable to that observed in older participants. These findings suggest that preserved compensatory mechanisms may still be engaged early after stroke and that VIM training, which directly stimulates the muscle spindle and engages sensorimotor and frontoparietal networks, may be highly relevant for older adults to limit age-related proprioceptive decline and to facilitate recovery after limb immobilization. Altogether, this cross-sectional study suggests potential implications for early rehabilitation after stroke and paves the way for future investigations into the long-term effects of VIM training on motor and cerebral function recovery in individuals with acute stroke.

## Supplementary Information


Supplementary Material 1.


## Data Availability

The fNIRS raw data are available on Recherche Data Gouv via the permanent link: https://doi.org/10.57745/HNZBV9.

## Abbreviations

BAs: Brodmann areas
EEG: Electroencephalography
FMA-UE: Fugl-Meyer Assessment for the Upper Extremity
fMRI: Functional magnetic resonance imaging
fNIRS: Functional near-infrared spectroscopy
HbO: Oxyhemoglobin
HbR: Deoxyhemoglobin
MNI: Montreal Neurological Institute
QT-NIRS: Quality testing of near infrared scans
SC: Short channel
SKIP: Standardized Kinesthetic Illusion Procedure
VIM: Vibration-induced illusion of movement

## References

[1] World Health Organization. Global strategy and action plan on ageing and health. Geneva: World Health Organization; 2017.

[2] The GBD 2016 Lifetime Risk of Stroke Collaborators. Global, Regional, and Country-Specific Lifetime Risks of Stroke, 1990 and 2016. N Engl J Med. 2018;379:2429–37. 10.1056/NEJMoa1804492 PMC6247346 30575491

[3] Pu L, Wang L, Zhang R, Zhao T, Jiang Y, Han L. Projected global trends in ischemic stroke incidence, deaths and disability-adjusted life years from 2020 to 2030. Stroke American Heart Association. 2023;54:1330–9. 10.1161/STROKEAHA.122.040073. 37094034

[4] Lawrence ES, Coshall C, Dundas R, Stewart J, Rudd AG, Howard R, et al. Estimates of the prevalence of acute stroke impairments and disability in a multiethnic population. Stroke American Heart Association. 2001;32:1279–84. 10.1161/01.STR.32.6.1279. 11387487

[5] Broeks GJ, Lankhorst GJ, Rumping K, Prevo AJH. The long-term outcome of arm function after stroke: results of a follow-up study. Disabil Rehabilitation. 1999;21:357–64. 10.1080/096382899297459. 10503976

[6] Connell L, Lincoln N, Radford K. Somatosensory impairment after stroke: frequency of different deficits and their recovery. Clin Rehabil. 2008;22:758–67. 10.1177/0269215508090674. 18678576

[7] Avanzino L, Pelosin E, Abbruzzese G, Bassolino M, Pozzo T, Bove M. Shaping motor cortex plasticity through proprioception. Cereb Cortex. 2014;24:2807–14. 10.1093/cercor/bht139. 23709641

[8] Roll R, Kavounoudias A, Albert F, Legré R, Gay A, Fabre B, et al. Illusory movements prevent cortical disruption caused by immobilization. Neuroimage. 2012;62:510–9. 10.1016/j.neuroimage.2012.05.016. 22584228

[9] Langer N, Hänggi J, Müller NA, Simmen HP, Jäncke L. Effects of limb immobilization on brain plasticity. Neurology. 2012;78:182–8. 10.1212/WNL.0b013e31823fcd9c. 22249495

[10] Yukawa Y, Higashi T, Minakuchi M, Naito E, Murata T. Vibration-induced illusory movement task can induce functional recovery in patients with subacute stroke. Cureus. 2024;16:e66667. 10.7759/cureus.66667. 39262538 PMC11388116

[11] Proske U, Gandevia SC. The proprioceptive senses: their roles in signaling body shape, body position and movement, and muscle force. Physiol Rev. 2012;92:1651–97. 10.1152/physrev.00048.2011. 23073629

[12] Desmurget M, Reilly KT, Richard N, Szathmari A, Mottolese C, Sirigu A. Movement intention after parietal cortex stimulation in humans. Science. 2009;324:811–3. 10.1126/science.1169896. 19423830

[13] Hagura N, Takei T, Hirose S, Aramaki Y, Matsumura M, Sadato N, et al. Activity in the posterior parietal cortex mediates visual dominance over kinesthesia. J Neurosci. 2007;27:7047–53. 10.1523/JNEUROSCI.0970-07.2007. 17596454 PMC6672236

[14] Naito E, Morita T, Amemiya K. Body representations in the human brain revealed by kinesthetic illusions and their essential contributions to motor control and corporeal awareness. Neurosci Res. 2016;104:16–30. 10.1016/j.neures.2015.10.013. 26562333

[15] Romaiguère P, Anton J-L, Roth M, Casini L, Roll J-P. Motor and parietal cortical areas both underlie kinaesthesia. Cogn Brain Res. 2003;16:74–82. 10.1016/S0926-6410(02)00221-5. 12589891

[16] Schneider C, Marquis R, Jöhr J, Lopes da Silva M, Ryvlin P, Serino A, et al. Disentangling the percepts of illusory movement and sensory stimulation during tendon vibration in the EEG. Neuroimage. 2021;241:118431. 10.1016/j.neuroimage.2021.118431. 34329723

[17] Roll JP, Vedel JP, Ribot E. Alteration of proprioceptive messages induced by tendon vibration in man: a microneurographic study. Exp Brain Res. 1989. 10.1007/BF00253639. 2753103

[18] Roll JP, Vedel JP. Kinaesthetic role of muscle afferents in man, studied by tendon vibration and microneurography. Exp Brain Res. 1982. 10.1007/BF00239377. 6214420

[19] Guerraz M, Provost S, Narison R, Brugnon A, Virolle S, Bresciani J-P. Integration of visual and proprioceptive afferents in kinesthesia. Neuroscience. 2012;223:258–68. 10.1016/j.neuroscience.2012.07.059. 22864182

[20] Lackner JR, Taublieb AB. Influence of vision on vibration-induced illusions of limb movement. Exp Neurol. 1984;85:97–106. 10.1016/0014-4886(84)90164-X. 6734787

[21] Naito E, Roland PE, Grefkes C, Choi HJ, Eickhoff S, Geyer S, et al. Dominance of the right hemisphere and role of area 2 in human kinesthesia. J Neurophysiol. 2005;93:1020–34. 10.1152/jn.00637.2004. 15385595

[22] Naito E, Ehrsson HH. Kinesthetic illusion of wrist movement activates motor-related areas. NeuroReport. 2001;12:3805–9. 10.1097/00001756-200112040-00041. 11726799

[23] Amemiya K, Naito E. Importance of human right inferior frontoparietal network connected by inferior branch of superior longitudinal fasciculus tract in corporeal awareness of kinesthetic illusory movement. Cortex. 2016;78:15–30. 10.1016/j.cortex.2016.01.017. 26986838

[24] Landelle C, Anton J-L, Nazarian B, Sein J, Gharbi A, Felician O, et al. Functional brain changes in the elderly for the perception of hand movements: a greater impairment occurs in proprioception than touch. Neuroimage. 2020;220:117056. 10.1016/j.neuroimage.2020.117056. 32562781

[25] Cignetti F, Vaugoyeau M, Nazarian B, Roth M, Anton J-L, Assaiante C. Boosted activation of right inferior frontoparietal network: a basis for illusory movement awareness. Hum Brain Mapp. 2014;35:5166–78. 10.1002/hbm.22541. 24798824 PMC6869717

[26] Ferrari F, Shell CE, Thumser ZC, Clemente F, Plow EB, Cipriani C, et al. Proprioceptive augmentation with illusory kinaesthetic sensation in stroke patients improves movement quality in an active upper limb reach-and-point task. Front Neurorobot. 2021. 10.3389/fnbot.2021.610673. 33732129 PMC7956990

[27] Naito E, Matsumoto R, Hagura N, Oouchida Y, Tomimoto H, Hanakawa T. Importance of precentral motor regions in human kinesthesia: a single case study. Neurocase. 2011;17:133–47. 10.1080/13554794.2010.498428. 20830645

[28] Khan F, Amatya B, Galea MP, Gonzenbach R, Kesselring J. Neurorehabilitation: applied neuroplasticity. J Neurol. 2017;264:603–15. 10.1007/s00415-016-8307-9. 27778158

[29] Viganò A, Celletti C, Giuliani G, Jannini TB, Marenco F, Maestrini I, et al. Focal muscle vibration (fMV) for post-stroke motor recovery: multisite neuroplasticity induction, timing of intervention, clinical approaches, and prospects from a narrative review. Vibration. 2023;6:645–58. 10.3390/vibration6030040.

[30] Huo C, Xu G, Xie H, Chen T, Shao G, Wang J, et al. Functional near-infrared spectroscopy in non-invasive neuromodulation. Neural Regen Res. 2024;19:1517. 10.4103/1673-5374.387970. 38051894 PMC10883499

[31] Anwar AR, Muthalib M, Perrey S, Galka A, Granert O, Wolff S, et al. Effective connectivity of cortical sensorimotor networks during finger movement tasks: a simultaneous fNIRS, fMRI, EEG study. Brain Topogr. 2016;29:645–60. 10.1007/s10548-016-0507-1. 27438589

[32] Hoshi Y. Hemodynamic signals in fNIRS. Prog Brain Res. 2016;225:153–79. 10.1016/bs.pbr.2016.03.004. 27130415

[33] Scholkmann F, Wolf M. General equation for the differential pathlength factor of the frontal human head depending on wavelength and age. J Biomed Opt. 2013;18:105004. 10.1117/1.JBO.18.10.105004. 24121731

[34] Yang L, Wang Z. Applications and advances of combined fMRI-fNIRs techniques in brain functional research. Front Neurol. 2025. 10.3389/fneur.2025.1542075. 40170894 PMC11958174

[35] Li R, Yang D, Fang F, Hong K-S, Reiss AL, Zhang Y. Concurrent fNIRS and EEG for brain function investigation: a systematic, methodology-focused review. Sensors. 2022;22:5865. 10.3390/s22155865. 35957421 PMC9371171

[36] Herold F, Wiegel P, Scholkmann F, Thiers A, Hamacher D, Schega L. Functional near-infrared spectroscopy in movement science: a systematic review on cortical activity in postural and walking tasks. Neurophotonics. 2017;4:041403. 10.1117/1.NPh.4.4.041403. 28924563 PMC5538329

[37] von Elm E, Altman DG, Egger M, Pocock SJ, Gøtzsche PC, Vandenbroucke JP, et al. The strengthening the reporting of observational studies in epidemiology (STROBE) statement: guidelines for reporting observational studies. PLOS Med. 2007;4:e296. 10.1371/journal.pmed.0040296. 17941714 PMC2020495

[38] Léger B, Auzou P, Fourdrinoy É, Sarrazin M, Celot S, Gay C, et al. Vibration-induced illusion of movement is hindered by acute stroke but mostly by aging: a cross-sectional study. Aging Clin Exp Res. 2025;38:20. 10.1007/s40520-025-03247-6. 41324779 PMC12775077

[39] Brott T, Adams HP, Olinger CP, Marler JR, Barsan WG, Biller J, et al. Measurements of acute cerebral infarction: a clinical examination scale. Stroke American Heart Association. 1989;20:864–70. 10.1161/01.STR.20.7.864. 2749846

[40] Fugl-Meyer A, Jääskö L, Leyman I, Olsson S, Steglind S. The post-stroke hemiplegic patient. 1. A method for evaluation of physical performance. JRM. 1975;7:13–31. 10.2340/1650197771331. 1135616

[41] Gauthier L, Dehaut F, Joanette Y. The bells test: a quantitative and qualitative test for visual neglect. Int J Clin Neuropsychol. 1989;11:49–54.

[42] Friedman PJ. Spatial neglect in acute stroke: the line bisection test. Scand J Rehabil Med. 1990;22:101–6. 2363023

[43] Oldfield RC. The assessment and analysis of handedness: the Edinburgh inventory. Neuropsychologia. 1971;9:97–113. 10.1016/0028-3932(71)90067-4. 5146491

[44] Otaka E, Otaka Y, Kasuga S, Nishimoto A, Yamazaki K, Kawakami M, et al. Reliability of the thumb localizing test and its validity against quantitative measures with a robotic device in patients with hemiparetic stroke. PLoS ONE. 2020;15:e0236437. 10.1371/journal.pone.0236437. 32706817 PMC7380647

[45] Rabin E, Gordon AM. Influence of fingertip contact on illusory arm movements. J Appl Physiol. 2004;96:1555–60. 10.1152/japplphysiol.01085.2003. 14698993

[46] Taylor MW, Taylor JL, Seizova-Cajic T. Muscle vibration-induced illusions: review of contributing factors, taxonomy of illusions and user’s guide. Multisens Res. 2017;30:25–63. 10.1163/22134808-00002544.

[47] Yücel MA, Anderson JE, Rogers D, Hajirahimi P, Farzam P, Gao Y, et al. Quantifying the impact of hair and skin characteristics on fNIRS signal quality for enhanced inclusivity. Nat Hum Behav. 2025. 10.1038/s41562-025-02274-7. 40897802 PMC13264803

[48] Yücel MA, Selb J, Aasted CM, Lin P-Y, Borsook D, Becerra L, et al. Mayer waves reduce the accuracy of estimated hemodynamic response functions in functional near-infrared spectroscopy. Biomed Opt Express. 2016;7:3078–88. 10.1364/BOE.7.003078. 27570699 PMC4986815

[49] Yücel MA, Lühmann AV, Scholkmann F, Gervain J, Dan I, Ayaz H, et al. Best practices for fNIRS publications. Neurophoton. 2021. 10.1117/1.NPh.8.1.012101. PMC7793571 33442557

[50] Beaulieu L-D, Schneider C, Massé-Alarie H, Ribot-Ciscar E. A new method to elicit and measure movement illusions in stroke by means of muscle tendon vibration: the Standardized Kinesthetic Illusion Procedure (SKIP). Somatosens Mot Res. 2020;37:28–36. 10.1080/08990220.2020.1713739. 31973656

[51] Ye JC, Tak S, Jang KE, Jung J, Jang J. NIRS-SPM: statistical parametric mapping for near-infrared spectroscopy. Neuroimage. 2009;44:428–47. 10.1016/j.neuroimage.2008.08.036. 18848897

[52] Aasted CM, Yücel MA, Cooper RJ, Dubb J, Tsuzuki D, Becerra L, et al. Anatomical guidance for functional near-infrared spectroscopy: AtlasViewer Tutorial. NPh. 2015;2:020801. 10.1117/1.NPh.2.2.020801. PMC4478785 26157991

[53] Pollonini L, Bortfeld H, Oghalai JS. PHOEBE: a method for real time mapping of optodes-scalp coupling in functional near-infrared spectroscopy. Biomed Opt Express. 2016;7:5104–19. 10.1364/BOE.7.005104. 28018728 PMC5175555

[54] Delaire É, Vincent T, Cai Z, Machado A, Hugueville L, Schwartz D, et al. NIRSTORM: a Brainstorm extension dedicated to functional near-infrared spectroscopy data analysis, advanced 3D reconstructions, and optimal probe design. NPh. 2025;12:025011. 10.1117/1.NPh.12.2.025011. PMC12081164 40375973

[55] Fishburn FA, Ludlum RS, Vaidya CJ, Medvedev AV. Temporal derivative distribution repair (TDDR): a motion correction method for fNIRS. Neuroimage. 2019;184:171–9. 10.1016/j.neuroimage.2018.09.025. 30217544 PMC6230489

[56] Pinti P, Scholkmann F, Hamilton A, Burgess P, Tachtsidis I. Current status and issues regarding pre-processing of fNIRS neuroimaging data: an investigation of diverse signal filtering methods within a general linear model framework. Front Hum Neurosci. 2019;12:505. 10.3389/fnhum.2018.00505. 30687038 PMC6336925

[57] Santosa H, Zhai X, Fishburn F, Sparto PJ, Huppert TJ. Quantitative comparison of correction techniques for removing systemic physiological signal in functional near-infrared spectroscopy studies. NPh. 2020;7:035009. 10.1117/1.NPh.7.3.035009. PMC7511246 32995361

[58] Franceschini MA, Joseph DK, Huppert TJ, Diamond SG, Boas DA. Diffuse optical imaging of the whole head. JBO. 2006;11:054007. 10.1117/1.2363365. 17092156 PMC2637816

[59] Scholkmann F, Tachtsidis I, Wolf M, Wolf U. Systemic physiology augmented functional near-infrared spectroscopy: a powerful approach to study the embodied human brain. Neurophotonics. 2022;9:030801. 10.1117/1.NPh.9.3.030801. 35832785 PMC9272976

[60] Delpy DT, Cope M, van der Zee P, Arridge S, Wray S, Wyatt J. Estimation of optical pathlength through tissue from direct time of flight measurement. Phys Med Biol. 1988;33:1433. 10.1088/0031-9155/33/12/008. 3237772

[61] Kinder KT, Heim HLR, Parker J, Lowery K, McCraw A, Eddings RN, et al. Systematic review of fNIRS studies reveals inconsistent chromophore data reporting practices. NPh. 2022;9:040601. 10.1117/1.NPh.9.4.040601. PMC9780687 36578778

[62] Tachtsidis I, Scholkmann F. False positives and false negatives in functional near-infrared spectroscopy: issues, challenges, and the way forward. Neurophotonics. 2016;3:030401. 10.1117/1.NPh.3.3.030401. PMC4791590 27054143

[63] Yang C-L, Lim SB, Peters S, Eng JJ. Cortical activation during shoulder and finger movements in healthy adults: a functional near-infrared spectroscopy (fNIRS) study. Front Hum Neurosci. 2020;14:260. 10.3389/fnhum.2020.00260. 32733221 PMC7362764

[64] Cui X, Bray S, Bryant DM, Glover GH, Reiss AL. A quantitative comparison of NIRS and fMRI across multiple cognitive tasks. Neuroimage. 2011;54:2808–21. 10.1016/j.neuroimage.2010.10.069. 21047559 PMC3021967

[65] Dravida S, Noah JA, Zhang X, Hirsch J. Comparison of oxyhemoglobin and deoxyhemoglobin signal reliability with and without global mean removal for digit manipulation motor tasks. NPh. 2017;5:011006. 10.1117/1.NPh.5.1.011006. PMC5597778 28924566

[66] Strangman G, Culver JP, Thompson JH, Boas DA. A quantitative comparison of simultaneous BOLD fMRI and NIRS recordings during functional brain activation. Neuroimage. 2002;17:719–31. 12377147

[67] Haeussinger FB, Dresler T, Heinzel S, Schecklmann M, Fallgatter AJ, Ehlis A-C. Reconstructing functional near-infrared spectroscopy (fNIRS) signals impaired by extra-cranial confounds: an easy-to-use filter method. Neuroimage. 2014;95:69–79. 10.1016/j.neuroimage.2014.02.035. 24657779

[68] Huppert TJ, Hoge RD, Diamond SG, Franceschini MA, Boas DA. A temporal comparison of BOLD, ASL, and NIRS hemodynamic responses to motor stimuli in adult humans. Neuroimage. 2006;29:368–82. 10.1016/j.neuroimage.2005.08.065. 16303317 PMC2692693

[69] Kirilina E, Jelzow A, Heine A, Niessing M, Wabnitz H, Brühl R, et al. The physiological origin of task-evoked systemic artefacts in functional near infrared spectroscopy. Neuroimage. 2012;61:70–81. 10.1016/j.neuroimage.2012.02.074. 22426347 PMC3348501

[70] Klein F, Debener S, Witt K, Kranczioch C. fMRI-based validation of continuous-wave fNIRS of supplementary motor area activation during motor execution and motor imagery. Sci Rep. 2022;12:3570. 10.1038/s41598-022-06519-7. 35246563 PMC8897516

[71] Chancel M, Landelle C, Blanchard C, Felician O, Guerraz M, Kavounoudias A. Hand movement illusions show changes in sensory reliance and preservation of multisensory integration with age for kinaesthesia. Neuropsychologia. 2018;119:45–58. 10.1016/j.neuropsychologia.2018.07.027. 30063911

[72] Landelle C, El Ahmadi A, Kavounoudias A. Age-related impairment of hand movement perception based on muscle proprioception and touch. Neuroscience. 2018;381:91–104. 10.1016/j.neuroscience.2018.04.015. 29684506

[73] Norman G. Likert scales, levels of measurement and the “laws” of statistics. Adv Health Sci Educ. 2010;15:625–32. 10.1007/s10459-010-9222-y. 20146096

[74] Driscoll WC. Robustness of the ANOVA and Tukey-Kramer statistical tests. Comput Ind Eng. 1996;31:265–8. 10.1016/0360-8352(96)00127-1.

[75] Benjamini Y, Hochberg Y. Controlling the false discovery rate: a practical and powerful approach to multiple testing. J R Stat Soc Ser B Stat Methodol. 1995;57:289–300. 10.1111/j.2517-6161.1995.tb02031.x.

[76] Cohen J. A power primer. Psychol Bull US. 1992;112:155–9. 10.1037/0033-2909.112.1.155. 19565683

[77] Kelly AMC, Garavan H. Human functional neuroimaging of brain changes associated with practice. Cereb Cortex. 2005;15:1089–102. 10.1093/cercor/bhi005. 15616134

[78] Muller CO, Faity G, Muthalib M, Perrey S, Dray G, Xu B, et al. Brain-movement relationship during upper-limb functional movements in chronic post-stroke patients. J NeuroEngineering Rehabil. 2024;21:188. 10.1186/s12984-024-01461-3. PMC11494975 39438994

[79] Golaszewski S, Frey V, Thomschewski A, Sebastianelli L, Versace V, Saltuari L, et al. Neural mechanisms underlying the Rubber Hand Illusion: a systematic review of related neurophysiological studies. Brain Behav. 2021;11:e02124. 10.1002/brb3.2124. 34288558 PMC8413782

[80] Chancel M, Brun C, Kavounoudias A, Guerraz M. The kinaesthetic mirror illusion: how much does the mirror matter? Exp Brain Res. 2016;234:1459–68. 10.1007/s00221-015-4549-5. 26790422

[81] Le Franc S, Fleury M, Cogne M, Butet S, Barillot C, Lecuyer A, et al. Influence of virtual reality visual feedback on the illusion of movement induced by tendon vibration of wrist in healthy participants. PLoS ONE. 2020;15:e0242416. 10.1371/journal.pone.0242416. 33216756 PMC7678999

[82] Fink GR, Marshall JC, Halligan PW, Frith CD, Driver J, Frackowiak RSJ, et al. The neural consequences of conflict between intention and the senses. Brain. 1999;122:497–512. 10.1093/brain/122.3.497. 10094258

[83] Wasaka T, Kakigi R. Conflict caused by visual feedback modulates activation in somatosensory areas during movement execution. Neuroimage. 2012;59:1501–7. 10.1016/j.neuroimage.2011.08.024. 21889595

[84] Zito GA, Wiest R, Aybek S. Neural correlates of sense of agency in motor control: a neuroimaging meta-analysis. PLoS ONE. 2020;15:e0234321. 10.1371/journal.pone.0234321. 32502189 PMC7274441

[85] Marly A, Yazdjian A, Soto-Faraco S. The role of conflict processing in multisensory perception: behavioural and electroencephalography evidence. Philos Trans R Soc Lond B Biol Sci. 2023;378:20220346. 10.1098/rstb.2022.0346. 37545310 PMC10404919

[86] Mastria G, Bertoni T, Perrin H, Akulenko N, Risso G, Akselrod M, et al. Body ownership alterations in stroke emerge from reduced proprioceptive precision and damage to the frontoparietal network. Med. 2024. 10.1016/j.medj.2024.10.013. 39532102

[87] Goble DJ, Brown SH. Upper limb asymmetries in the matching of proprioceptive versus visual targets. J Neurophysiol Am Physiol Soc. 2008;99:3063–74. 10.1152/jn.90259.2008. 18436632

[88] Goble DJ, Brown SH. The biological and behavioral basis of upper limb asymmetries in sensorimotor performance. Neurosci Biobehav Rev. 2008;32:598–610. 10.1016/j.neubiorev.2007.10.006. 18160103

[89] Sainburg RL. Chapter 8 - laterality of basic motor control mechanisms: different roles of the right and left brain hemispheres. In: Loffing F, Hagemann N, Strauss B, MacMahon C, editors. Laterality in sports. San Diego: Academic Press; 2016. p. 155–77. 10.1016/B978-0-12-801426-4.00008-0.

[90] Cabeza R, Albert M, Belleville S, Craik FIM, Duarte A, Grady CL, et al. Maintenance, reserve and compensation: the cognitive neuroscience of healthy ageing. Nat Rev Neurosci. 2018;19:701–10. 10.1038/s41583-018-0068-2. 30305711 PMC6472256

[91] Reuter-Lorenz PA, Cappell KA. Neurocognitive aging and the compensation hypothesis. Curr Dir Psychol Sci. 2008;17:177–82. 10.1111/j.1467-8721.2008.00570.x.

[92] Sunnerhagen KS, Opheim A, Alt Murphy M. Onset, time course and prediction of spasticity after stroke or traumatic brain injury. Ann Phys Rehabil Med. 2019;62:431–4. 10.1016/j.rehab.2018.04.004. 29753889

[93] Amiez N, Beaulieu L-D, Paizis C, Lapole T, Rienzo FD. Muscle tendon vibration revisited: Why tonic vibration reflex and kinaesthetic illusions matter. J Physiol. 2026;604:3231–52. 10.1113/JP290085. 41902685 PMC13082191

[94] Alia C, Spalletti C, Lai S, Panarese A, Lamola G, Bertolucci F, et al. Neuroplastic changes following brain ischemia and their contribution to stroke recovery: novel approaches in neurorehabilitation. Front Cell Neurosci. 2017. 10.3389/fncel.2017.00076. 28360842 PMC5352696

[95] Premilovac D, Sutherland BA. Acute and long-term changes in blood flow after ischemic stroke: challenges and opportunities. Neural Regen Res. 2023;18:799. 10.4103/1673-5374.350699. 36204841 PMC9700091

[96] Honda K, Nakashima Y, Hua C, Yamamoto M. Influence of combined vibration and electrical stimulation on latency of kinesthetic illusion. JRM. 2023;35:823–33. 10.20965/jrm.2023.p0823.

[97] Lin L, Qing W, Huang Y, Ye F, Rong W, Li W, et al. Comparison of immediate neuromodulatory effects between focal vibratory and electrical sensory stimulations after stroke. Bioengineering (Basel). 2024;11:286. 10.3390/bioengineering11030286. 38534560 PMC10967870

[98] Muthalib M, Re R, Zucchelli L, Perrey S, Contini D, Caffini M, et al. Effects of increasing neuromuscular electrical stimulation current intensity on cortical sensorimotor network activation: a time domain fNIRS study. PLoS ONE. 2015;10:e0131951. 10.1371/journal.pone.0131951. 26158464 PMC4497661

[99] Rangwani R, Park H. A new approach of inducing proprioceptive illusion by transcutaneous electrical stimulation. J Neuroeng Rehabil. 2021;18:73. 10.1186/s12984-021-00870-y. 33941209 PMC8094608

[100] Le Franc S, Fleury M, Jeunet C, Butet S, Barillot C, Bonan I, et al. Influence of the visuo-proprioceptive illusion of movement and motor imagery of the wrist on EEG cortical excitability among healthy participants. PLoS ONE. 2021;16:e0256723. 10.1371/journal.pone.0256723. 34473788 PMC8412266

[101] Rinderknecht MD, Yeongmi Kim, Santos-Carreras L, Bleuler H, Gassert R. Combined tendon vibration and virtual reality for post-stroke hand rehabilitation. In: 2013 World haptics conference (WHC). Daejeon: IEEE; 2013. pp 277–82. 10.1109/WHC.2013.6548421

[102] Cabeza R. Hemispheric asymmetry reduction in older adults: The HAROLD model. Psychol Aging. 2002;17:85–100. 10.1037/0882-7974.17.1.85. 11931290

[103] Acosta-Sojo Y, Martin BJ. Age-related differences in proprioceptive asymmetries. Neurosci Lett. 2021;757:135992. 10.1016/j.neulet.2021.135992. 34051338

[104] Kawai-Takaishi M, Hosoyama T. Muscle spindle afferent neurons preferentially degenerate with aging. Sci Rep. 2025;15:23946. 10.1038/s41598-025-08270-1. 40615509 PMC12227564

[105] Kröger S, Watkins B. Muscle spindle function in healthy and diseased muscle. Skelet Muscle. 2021;11:3. 10.1186/s13395-020-00258-x. 33407830 PMC7788844

[106] Goble DJ, Coxon JP, Wenderoth N, Van Impe A, Swinnen SP. Proprioceptive sensibility in the elderly: degeneration, functional consequences and plastic-adaptive processes. Neurosci Biobehav Rev. 2009;33:271–8. 10.1016/j.neubiorev.2008.08.012. 18793668

[107] Carradus AJ, Mougin O, Hunt BAE, Tewarie PK, Geades N, Morris PG, et al. Age-related differences in myeloarchitecture measured at 7 T. Neurobiol Aging. 2020;96:246–54. 10.1016/j.neurobiolaging.2020.08.009. 33049517

[108] Hutton RS, Atwater SW. Acute and chronic adaptations of muscle proprioceptors in response to increased use. Sports Med. 1992;14:406–21. 10.2165/00007256-199214060-00007. 1470793

[109] Seene T, Kaasik P. Biological characteristics of structural and functional remodelling in skeletal muscle: effect of exercise. asb. 2013;5:251–78. 10.12988/asb.2013.327.

[110] Ackerley R, Samain-Aupic L, Ribot-Ciscar E. Passive proprioceptive training alters the sensitivity of muscle spindles to imposed movements. eNeuro. 2022. 10.1523/ENEURO.0249-21.2021. 35022185 PMC8805769

[111] Muthukrishnan P, Shanmugalakshmi G. Neurophysiological impact of static stretching on cerebral activity: a comprehensive evidence-based analysis of proprioceptive integration, motor cortical activation, and epigenetic modulation. Rochester, NY: Social Science Research Network; 2025. 10.2139/ssrn.5826042.

[112] Voss MW, Prakash RS, Erickson KI, Basak C, Chaddock L, Kim JS, et al. Plasticity of brain networks in a randomized intervention trial of exercise training in older adults. Front Aging Neurosci. 2010. 10.3389/fnagi.2010.00032. 20890449 PMC2947936

[113] Domingos C, Pêgo JM, Santos NC. Effects of physical activity on brain function and structure in older adults: a systematic review. Behav Brain Res. 2021;402:113061. 10.1016/j.bbr.2020.113061. 33359570

[114] Adamo DE, Alexander NB, Brown SH. The influence of age and physical activity on upper limb proprioceptive ability. J Aging Phys Act. 2009;17:272–93. 10.1123/japa.17.3.272. 19799100

[115] Ribeiro F, Oliveira J. Aging effects on joint proprioception: the role of physical activity in proprioception preservation. Eur Rev Aging Phys Act. 2007;4:71–6. 10.1007/s11556-007-0026-x.

[116] Van de Winckel A, Tseng Y-T, Chantigian D, Lorant K, Zarandi Z, Buchanan J, et al. Age-related decline of wrist position sense and its relationship to specific physical training. Front Hum Neurosci. 2017. 10.3389/fnhum.2017.00570. 29209188 PMC5702425

[117] Wei X, Sun S, Zhang M, Zhao Z. A systematic review and meta-analysis of clinical efficacy of early and late rehabilitation interventions for ischemic stroke. BMC Neurol. 2024;24:91. 10.1186/s12883-024-03565-8. 38459477 PMC10921695

[118] Shurupova M, Fuchizhi O, Ivanova G. Greater recovery of upper-limb function in stroke patients with repetitive functional proprioceptive stimulation compared to robotic mechanotherapy: a quasi-randomized controlled trial. BMC Sports Sci Med Rehabil. 2026;18:79. 10.1186/s13102-025-01526-3. 41555385 PMC12895587

[119] Leff DR, Orihuela-Espina F, Elwell CE, Athanasiou T, Delpy DT, Darzi AW, et al. Assessment of the cerebral cortex during motor task behaviours in adults: a systematic review of functional near infrared spectroscopy (fNIRS) studies. Neuroimage. 2011;54:2922–36. 10.1016/j.neuroimage.2010.10.058. 21029781

[120] Schroeter ML, Kupka T, Mildner T, Uludağ K, von Cramon DY. Investigating the post-stimulus undershoot of the BOLD signal—a simultaneous fMRI and fNIRS study. Neuroimage. 2006;30:349–58. 10.1016/j.neuroimage.2005.09.048. 16257236

