# Supplementary material for: Cortical neuromodulation by vibration-induced illusion of movement is observed in older individuals, but not in individuals with acute stroke: a cross-sectional fNIRS study
Source: J Neuroeng Rehabil. 2026 Aug 31;23:254. doi: 10.1186/s12984-026-02129-w (PMC13528179; doi:10.1186/s12984-026-02129-w)
Supplement: Supplementary file 1 [file 12984_2026_2129_MOESM1_ESM.docx]

**Supplementary materials**

Table 1: Functional localization of lesions in acute stroke patients

| **Patients** | **S1** | **M1** | **PMC** | **SMA** | **CSF** | **V1** |
| --- | --- | --- | --- | --- | --- | --- |
| 1 |  |  |  |  | X |  |
| 2 |  |  |  |  | X |  |
| 3 |  |  |  |  | X |  |
| 4 | X | X | X |  |  |  |
| 5 |  |  |  |  |  |  |
| 6 | X | X | X |  | X |  |
| 7 |  |  |  |  |  |  |
| 8 |  |  |  |  | X |  |
| 9 |  |  | X | X |  |  |
| 10 | X | X |  |  |  |  |
| 11 |  | X |  |  | X |  |
| 12 |  |  |  |  |  |  |
| 13 |  |  |  |  | X |  |
| 14 |  |  |  |  | X |  |
| 15 |  |  |  |  | X |  |
| 16 |  |  |  |  |  |  |
| 17 |  |  |  |  | X |  |
| 18 | X | X |  |  |  | X |
| 19 |  |  | X |  | X |  |
| 20 | X | X | X |  |  | X |
| 21 |  |  |  |  |  |  |
| 22 |  | X |  |  |  |  |
| 23 |  |  |  |  | X |  |
| ***CSF****: Corticospinal fasciculus;* ***M1****: Primary Motor cortex;* ***PMC****: Premotor Cortex;* ***S1****: Primary Somatosensory area;* ***SMA****: Supplementary Motor Area;* ***V1****: Primary visual cortex* | | | | | | |

Table 2: Anatomical localization of lesions in acute stroke patients

| **Patients** | **CN** | **LN** | **IC** | **EC** | **Th** | **CR** | **Ins** | **FC** | **PC** | **OC** | **P/Bstem** |
| --- | --- | --- | --- | --- | --- | --- | --- | --- | --- | --- | --- |
| 1 |  |  |  |  |  | X |  |  |  |  | X |
| 2 |  |  |  |  |  | X |  |  |  |  |  |
| 3 |  |  | X |  |  |  |  |  |  |  |  |
| 4 |  |  |  |  |  |  |  | X | X |  |  |
| 5 |  | X |  |  |  |  |  |  |  |  |  |
| 6 | X | X | X | X |  | X | X | X | X |  |  |
| 7 | X | X | X | X |  | X | X |  |  |  |  |
| 8 |  |  | X |  | X |  |  |  |  |  |  |
| 9 | X | X |  |  |  |  |  | X |  |  |  |
| 10 |  |  |  |  |  |  | X | X | X |  |  |
| 11 | X | X | X | X | X | X | X | X |  |  |  |
| 12 | X | X |  | X |  |  |  |  |  |  |  |
| 13 |  |  | X |  | X |  |  |  |  |  |  |
| 14 | X | X | X |  |  |  |  |  |  |  |  |
| 15 |  | X | X |  |  |  |  |  |  |  |  |
| 16 |  | X |  |  |  |  | X | X |  |  |  |
| 17 | X | X |  |  |  |  |  |  |  |  |  |
| 18 |  |  | X |  |  |  | X | X | X | X |  |
| 19 | X | X | X | X |  |  | X | X |  |  |  |
| 20 | X |  |  |  |  | X |  | X | X | X |  |
| 21 | X |  |  |  |  |  |  |  |  |  |  |
| 22 |  |  |  |  |  |  |  | X | X |  |  |
| 23 |  |  |  |  |  |  |  |  |  |  | X |
| ***CN****: Caudate Nucleus;* ***CR****: Corona Radiata;* ***EC****: External Capsule;* ***FC****: Frontal Cortex;* ***IC****: Internal Capsule;* ***Ins****: Insula;* ***LN****: Lenticular Nucleus;* ***P/Bstem****: Pons or Brain stem;* ***PC****: Parietal Cortex;* ***OC****: Occipital Cortex;* ***Th****: Thalamus* | | | | | | | | | | | |

Table 3: Baseline characteristics comparisons between each group. Sex was compared between each group using Pearson's Chi-squared tests with Yates' continuity correction. Other characteristics were compared with a one-way ANOVA followed by post-hoc Tukey-Kramer’s tests when applicable. Right TLT was compared using a Student’s t-test.

|  | Young - Older - Stroke | Young - Older | Young - Stroke | Older - Stroke |
| --- | --- | --- | --- | --- |
| Sex | - | X² = 0  p = 1 | X² = 0.65  p = 0.42 | X² = 0.35  p = 0.56 |
| Handedness Laterality Index | F(2,78) = 2.05  p = 0.14 | - | - | - |
| Channels rejected | F(2,78) = 8.70  p < 0.001 | p < 0.001 | p = 0.08 | p = 0.19 |
| Differential Pathlength Factors |  |  |  |  |
| 760 nm | F(2,78) = 210.42  p < 0.001 | p < 0.001 | p < 0.001 | p = 0.19 |
| 842 nm | F(2,78) = 210.42  p < 0.001 | p < 0.001 | p < 0.001 | p = 0.19 |
| Thumb Localizing Test |  |  |  |  |
| Left | F(2,78) = 11.11  p < 0.001 | p = 1.00 | p < 0.001 | p < 0.001 |
| Right | - | p = 1.00 | - | - |

Table 4: Channel locations and statistical results (HbO/HbR FDR-corrected p-values and Hedges’ g) for each group and condition

| ***Conditions*** |  | Young | | | | | |  |  | Older | | | | | |  |  | Acute stroke | |
| --- | --- | --- | --- | --- | --- | --- | --- | --- | --- | --- | --- | --- | --- | --- | --- | --- | --- | --- | --- |
| Arm |  | Left | |  |  | Right | |  |  | Left | |  |  | Right | |  |  | Left | |
| Vision |  | With | Without |  |  | With | Without |  |  | With | Without |  |  | With | Without |  |  | With | Without |
|  |  |  |  |  |  |  |  |  |  |  |  |  |  |  |  |  |  |  |  |
| ***Channels*** |  |  |  |  |  |  |  |  |  |  |  |  |  |  |  |  |  |  |  |
| *Frontal* |  |  |  |  |  |  |  |  |  |  |  |  |  |  |  |  |  |  |  |
| *Left* |  |  |  |  |  |  |  |  |  |  |  |  |  |  |  |  |  |  |  |
| 14 | HbO | 0.52 (0.16) | 0.83 (-0.08) |  |  | 0.98 (0.02) | 0.84 (0.11) |  |  | 0.09 (0.39) | 0.13 (0.33) |  |  | 0.15 (0.37) | **0.02* (0.49)** |  |  | 0.91 (-0.08) | 0.72 (0.22) |
|  | HbR | 0.24 (-0.31) | 0.56 (-0.24) |  |  | 0.14 (-0.41) | 0.29 (-0.29) |  |  | 0.95 (-0.01) | 0.73 (-0.07) |  |  | 0.94 (-0.01) | 0.13 (-0.34) |  |  | 0.45 (-0.28) | 0.88 (-0.21) |
| 16 | HbO | 0.24 (-0.29) | 0.50 (-0.26) |  |  | 0.82 (-0.17) | 0.71 (-0.24) |  |  | 0.11 (0.35) | 0.05 (0.44) |  |  | 0.30 (0.25) | 0.06 (0.40) |  |  | 0.91 (-0.06) | 0.95 (-0.04) |
|  | HbR | 0.83 (-0.05) | 0.78 (0.10) |  |  | 0.24 (0.28) | 0.67 (-0.11) |  |  | 0.63 (0.12) | 0.85 (0.03) |  |  | 0.87 (0.05) | 0.32 (0.22) |  |  | 0.91 (-0.04) | 0.52 (-0.41) |
| 17 | HbO | 0.17 (-0.35) | 0.65 (-0.15) |  |  | 0.82 (-0.18) | 0.84 (-0.12) |  |  | 0.07 (0.46) | 0.09 (0.37) |  |  | 0.47 (0.16) | 0.24 (0.24) |  |  | 0.84 (0.22) | 0.72 (0.20) |
|  | HbR | 0.50 (-0.17) | 0.78 (-0.11) |  |  | 0.46 (-0.19) | 0.29 (-0.27) |  |  | 0.64 (-0.11) | 0.43 (-0.16) |  |  | 0.87 (-0.05) | 0.18 (-0.29) |  |  | 0.45 (-0.35) | 0.88 (-0.19) |
| *Right* |  |  |  |  |  |  |  |  |  |  |  |  |  |  |  |  |  |  |  |
| 38 | HbO | 0.99 (0.00) | 0.83 (-0.07) |  |  | 0.98 (-0.04) | 0.84 (-0.12) |  |  | **0.01* (0.70)** | **0.01* (0.62)** |  |  | 0.65 (0.11) | **0.01* (0.67)** |  |  | 0.83 (0.36) | 0.95 (-0.03) |
|  | HbR | **0.03* (-0.58)** | 0.26 (-0.39) |  |  | 0.07 (-0.49) | 0.29 (-0.28) |  |  | 0.54 (-0.17) | 0.10 (-0.35) |  |  | 0.14 (-0.35) | **0.04* (-0.50)** |  |  | 0.49 (-0.24) | 0.52 (-0.39) |
| 41 | HbO | 0.14 (0.38) | 0.65 (0.15) |  |  | 0.23 (0.52) | 0.62 (0.31) |  |  | 0.08 (0.45) | **0.00* (0.86)** |  |  | 0.96 (-0.01) | **0.02* (0.54)** |  |  | 0.94 (0.03) | 0.72 (0.23) |
|  | HbR | **0.02* (-0.66)** | 0.06 (-0.59) |  |  | **0.00* (-0.84)** | 0.29 (-0.30) |  |  | 0.15 (-0.33) | **0.03* (-0.47)** |  |  | 0.11 (-0.37) | **0.03* (-0.52)** |  |  | 0.45 (-0.31) | 0.88 (-0.28) |
| 42 | HbO | 0.70 (-0.09) | 0.51 (-0.21) |  |  | 0.98 (-0.05) | 0.84 (-0.12) |  |  | 0.10 (0.38) | **0.03* (0.51)** |  |  | 1.00 (0.00) | 0.22 (0.26) |  |  | 0.91 (0.09) | 0.95 (0.08) |
|  | HbR | 0.67 (0.12) | 0.96 (-0.01) |  |  | 0.42 (0.21) | 0.65 (-0.12) |  |  | 0.95 (0.01) | 0.77 (-0.06) |  |  | 0.28 (-0.23) | 0.77 (-0.05) |  |  | 0.83 (-0.10) | 0.97 (-0.08) |
|  |  |  |  |  |  |  |  |  |  |  |  |  |  |  |  |  |  |  |  |
| *dlPFC* |  |  |  |  |  |  |  |  |  |  |  |  |  |  |  |  |  |  |  |
| *Left* |  |  |  |  |  |  |  |  |  |  |  |  |  |  |  |  |  |  |  |
| 18 | HbO | 0.63 (-0.12) | 0.50 (-0.26) |  |  | 0.98 (0.02) | 0.69 (-0.26) |  |  | 0.61 (0.13) | 0.32 (0.22) |  |  | 0.18 (0.32) | 0.34 (0.20) |  |  | 0.91 (-0.07) | 0.72 (0.20) |
|  | HbR | 0.17 (-0.35) | 0.79 (-0.08) |  |  | **0.05* (-0.56)** | 0.08 (-0.46) |  |  | 0.25 (-0.26) | 0.20 (-0.27) |  |  | 0.33 (-0.20) | 0.01 (-0.68) |  |  | 0.45 (-0.27) | 0.52 (-0.43) |
| *Right* |  |  |  |  |  |  |  |  |  |  |  |  |  |  |  |  |  |  |  |
| 37 | HbO | 0.12 (0.41) | 0.69 (0.13) |  |  | 0.85 (0.11) | 0.86 (0.09) |  |  | 0.22 (0.28) | 0.11 (0.35) |  |  | 0.71 (0.09) | 0.45 (0.16) |  |  | 0.97 (-0.01) | 0.72 (0.28) |
|  | HbR | **0.01* (-0.69)** | 0.61 (-0.19) |  |  | **0.05* (-0.56)** | 0.08 (-0.47) |  |  | **0.01* (-0.73)** | **0.00* (-0.64)** |  |  | 0.05 (-0.49) | **0.00* (-1.00)** |  |  | 0.08 (-0.68) | 0.52 (-0.39) |
|  |  |  |  |  |  |  |  |  |  |  |  |  |  |  |  |  |  |  |  |
| *Motor* |  |  |  |  |  |  |  |  |  |  |  |  |  |  |  |  |  |  |  |
| *Left* |  |  |  |  |  |  |  |  |  |  |  |  |  |  |  |  |  |  |  |
| 9 | HbO | 0.48 (-0.21) | 0.83 (-0.07) |  |  | 0.26 (0.49) | 0.72 (0.24) |  |  | 0.71 (0.09) | 0.25 (0.27) |  |  | 0.24 (0.29) | **0.02* (0.56)** |  |  | 0.91 (-0.05) | 0.95 (-0.05) |
|  | HbR | 0.24 (-0.35) | 0.90 (0.05) |  |  | 1.00 (0.00) | 0.62 (-0.16) |  |  | 0.09 (-0.45) | **0.00* (-0.68)** |  |  | **0.00* (-0.86)** | **0.04* (-0.54)** |  |  | 0.72 (0.17) | 0.97 (-0.10) |
| 19 | HbO | 0.20 (-0.32) | 0.47 (-0.31) |  |  | 0.26 (-0.41) | 0.41 (-0.43) |  |  | 0.66 (0.11) | 0.70 (0.10) |  |  | 0.30 (0.24) | **0.03* (0.47)** |  |  | 0.91 (0.04) | 0.95 (0.09) |
|  | HbR | 0.37 (-0.24) | 0.78 (-0.09) |  |  | 0.20 (-0.33) | 0.29 (-0.27) |  |  | 0.17 (-0.31) | **0.04* (-0.44)** |  |  | 0.30 (-0.22) | **0.04* (-0.49)** |  |  | 0.89 (0.05) | 0.97 (-0.04) |
| 20 | HbO | 0.63 (-0.11) | 0.65 (-0.17) |  |  | 0.85 (-0.15) | 0.84 (-0.14) |  |  | 0.81 (0.05) | 0.82 (0.07) |  |  | 0.15 (0.37) | **0.01* (0.63)** |  |  | 0.83 (-0.35) | 0.95 (-0.03) |
|  | HbR | 0.84 (0.05) | 0.67 (0.17) |  |  | 0.42 (-0.21) | 0.10 (-0.42) |  |  | 0.64 (-0.11) | 0.40 (-0.18) |  |  | 0.10 (-0.40) | 0.13 (-0.34) |  |  | 0.45 (-0.33) | 0.97 (0.05) |
| 21 | HbO | 0.96 (-0.02) | 0.34 (-0.39) |  |  | 0.85 (-0.13) | 0.72 (-0.24) |  |  | 0.61 (-0.13) | 0.90 (0.03) |  |  | 0.16 (0.34) | **0.02* (0.51)** |  |  | 0.83 (-0.31) | 0.95 (0.03) |
|  | HbR | 0.76 (-0.10) | 0.58 (0.21) |  |  | 0.70 (-0.11) | 0.47 (-0.21) |  |  | 0.13 (-0.36) | 0.43 (-0.16) |  |  | 0.05 (-0.47) | **0.01* (-0.64)** |  |  | 0.61 (-0.21) | 0.97 (-0.05) |
| 22 | HbO | 0.59 (-0.13) | 0.34 (-0.42) |  |  | 0.63 (-0.26) | 0.54 (-0.33) |  |  | 0.66 (-0.10) | 0.88 (0.05) |  |  | 0.09 (0.45) | **0.02* (0.52)** |  |  | 0.83 (-0.31) | 0.95 (-0.08) |
|  | HbR | 0.73 (0.10) | 0.56 (0.23) |  |  | 0.79 (-0.07) | 0.71 (-0.08) |  |  | 0.63 (0.12) | 0.71 (-0.08) |  |  | 0.11 (-0.36) | 0.22 (-0.26) |  |  | 0.91 (-0.03) | 0.88 (0.17) |
| 23 | HbO | 0.48 (-0.19) | 0.88 (-0.04) |  |  | 0.63 (0.23) | 0.84 (0.11) |  |  | 0.93 (-0.02) | 0.42 (0.18) |  |  | 0.11 (0.41) | **0.01* (0.61)** |  |  | 0.83 (-0.29) | 0.95 (-0.09) |
|  | HbR | 0.21 (-0.33) | 0.70 (0.16) |  |  | 0.46 (-0.20) | 0.11 (-0.41) |  |  | 0.95 (0.02) | 0.17 (-0.29) |  |  | 0.06 (-0.45) | **0.04* (-0.49)** |  |  | 0.65 (-0.19) | 0.88 (-0.24) |
| *Right* |  |  |  |  |  |  |  |  |  |  |  |  |  |  |  |  |  |  |  |
| 32 | HbO | 0.71 (0.10) | 0.51 (0.29) |  |  | 1.00 (0.00) | 0.69 (-0.32) |  |  | 0.09 (0.43) | **0.01* (0.69)** |  |  | 0.87 (-0.05) | 0.74 (0.08) |  |  | 0.84 (0.17) | 0.65 (0.44) |
|  | HbR | 0.17 (-0.46) | 0.56 (-0.28) |  |  | 0.64 (0.15) | 0.79 (0.07) |  |  | **0.01* (-0.80)** | **0.00* (-0.96)** |  |  | 0.05 (-0.54) | 0.13 (-0.38) |  |  | 0.97 (-0.01) | 0.88 (-0.26) |
| 39 | HbO | 0.63 (0.11) | 0.88 (0.03) |  |  | 0.56 (-0.28) | 0.72 (-0.20) |  |  | 0.09 (0.40) | **0.01* (0.64)** |  |  | 0.41 (-0.18) | 0.42 (0.17) |  |  | 0.97 (0.01) | 0.81 (0.16) |
|  | HbR | **0.05* (-0.51)** | 0.56 (-0.22) |  |  | 0.08 (-0.48) | 0.29 (-0.28) |  |  | **0.03* (-0.51)** | 0.08 (-0.36) |  |  | 0.24 (-0.26) | **0.04* (-0.47)** |  |  | 0.84 (-0.08) | 0.97 (-0.06) |
| 43 | HbO | 0.12 (0.41) | 0.88 (0.03) |  |  | 0.50 (-0.34) | 0.38 (-0.47) |  |  | 0.14 (0.33) | **0.05* (0.45)** |  |  | 0.39 (-0.20) | 0.80 (-0.06) |  |  | 0.84 (-0.17) | 0.73 (0.18) |
|  | HbR | 0.78 (-0.08) | 0.58 (-0.20) |  |  | 0.79 (0.06) | 0.71 (0.09) |  |  | **0.03* (-0.53)** | **0.00* (-0.79)** |  |  | 0.50 (-0.15) | 0.65 (0.10) |  |  | 0.73 (0.13) | 0.88 (0.15) |
| 44 | HbO | 0.49 (0.18) | 0.50 (0.28) |  |  | 0.26 (-0.46) | 0.53 (-0.38) |  |  | 0.14 (0.32) | 0.20 (0.28) |  |  | 0.64 (-0.12) | 0.80 (0.05) |  |  | 0.84 (-0.20) | 0.72 (0.23) |
|  | HbR | 0.08 (-0.51) | 0.79 (-0.09) |  |  | 0.82 (0.05) | 0.65 (0.13) |  |  | **0.02* (-0.61)** | **0.00* (-0.65)** |  |  | 0.64 (-0.11) | 0.18 (-0.28) |  |  | 0.45 (-0.28) | 0.97 (0.02) |
| 45 | HbO | 0.30 (0.31) | 0.34 (0.50) |  |  | 0.98 (-0.04) | 0.69 (-0.32) |  |  | 0.08 (0.43) | 0.10 (0.36) |  |  | 0.96 (0.02) | 0.71 (0.09) |  |  | 0.91 (0.05) | 0.72 (0.36) |
|  | HbR | 0.32 (-0.30) | 0.26 (-0.47) |  |  | 0.49 (0.20) | 0.70 (0.11) |  |  | 0.06 (-0.46) | **0.00* (-0.80)** |  |  | 0.14 (-0.34) | 0.18 (-0.29) |  |  | 0.91 (0.03) | 0.97 (0.04) |
| 46 | HbO | 0.48 (0.18) | 0.76 (0.10) |  |  | **0.01* (-0.78)** | 0.36 (-0.53) |  |  | 0.09 (0.40) | 0.05 (0.43) |  |  | 0.11 (-0.42) | 0.99 (0.00) |  |  | 0.84 (-0.21) | 0.95 (0.09) |
|  | HbR | 0.78 (-0.07) | 0.96 (0.01) |  |  | 0.77 (0.08) | 0.09 (0.43) |  |  | 0.15 (-0.33) | **0.01* (-0.56)** |  |  | 0.90 (-0.03) | 0.52 (0.15) |  |  | 0.80 (-0.10) | 0.97 (0.05) |
|  |  |  |  |  |  |  |  |  |  |  |  |  |  |  |  |  |  |  |  |
| *S1* |  |  |  |  |  |  |  |  |  |  |  |  |  |  |  |  |  |  |  |
| *Left* |  |  |  |  |  |  |  |  |  |  |  |  |  |  |  |  |  |  |  |
| 8 | HbO | 0.45 (0.20) | 0.65 (0.17) |  |  | 0.63 (0.24) | 0.72 (0.21) |  |  | 0.66 (0.10) | 0.20 (0.28) |  |  | 0.08 (0.50) | **0.01* (0.56)** |  |  | 0.96 (0.02) | 0.95 (0.05) |
|  | HbR | 0.78 (-0.07) | 0.78 (-0.09) |  |  | 0.12 (-0.44) | **0.01* (-0.74)** |  |  | 0.91 (-0.04) | 0.07 (-0.38) |  |  | 0.08 (-0.42) | 0.06 (-0.44) |  |  | 0.73 (-0.14) | 0.88 (0.19) |
| 11 | HbO | 0.20 (0.32) | 0.86 (-0.05) |  |  | 0.85 (-0.12) | 0.97 (0.03) |  |  | 0.54 (0.16) | 0.86 (0.05) |  |  | 0.08 (0.49) | **0.01* (0.58)** |  |  | 0.89 (-0.14) | 0.98 (0.01) |
|  | HbR | 0.45 (-0.20) | 0.73 (0.13) |  |  | 0.19 (-0.33) | **0.03* (-0.58)** |  |  | 0.18 (-0.30) | 0.06 (-0.40) |  |  | 0.05 (-0.48) | 0.32 (-0.22) |  |  | 0.08 (-0.65) | 0.97 (-0.01) |
| 13 | HbO | 0.71 (0.08) | 0.34 (-0.37) |  |  | 0.98 (-0.03) | 0.97 (-0.02) |  |  | 0.74 (0.07) | 0.70 (0.10) |  |  | 0.09 (0.46) | 0.07 (0.39) |  |  | 0.84 (-0.20) | 0.98 (0.01) |
|  | HbR | 0.84 (0.04) | 0.46 (0.32) |  |  | 0.15 (-0.38) | 0.08 (-0.47) |  |  | 0.56 (-0.15) | 0.11 (-0.32) |  |  | **0.03* (-0.56)** | 0.17 (-0.30) |  |  | 0.84 (0.07) | 0.88 (0.17) |
| *Right* |  |  |  |  |  |  |  |  |  |  |  |  |  |  |  |  |  |  |  |
| 31 | HbO | **0.02* (0.72)** | 0.34 (0.39) |  |  | 0.87 (-0.11) | 0.84 (-0.15) |  |  | 0.10 (0.37) | **0.01* (0.58)** |  |  | 0.17 (0.33) | 0.23 (0.25) |  |  | 0.91 (0.07) | 0.72 (0.36) |
|  | HbR | 0.49 (-0.19) | 0.56 (-0.27) |  |  | 0.49 (0.18) | 0.80 (0.05) |  |  | **0.03* (-0.52)** | **0.00* (-0.96)** |  |  | 0.24 (-0.26) | 0.61 (0.11) |  |  | 0.80 (0.11) | 0.97 (-0.07) |
| 34 | HbO | **0.05* (0.52)** | 0.65 (0.17) |  |  | 0.85 (-0.12) | 0.84 (-0.12) |  |  | **0.01* (0.65)** | **0.04* (0.46)** |  |  | 0.18 (0.32) | 0.21 (0.27) |  |  | 0.91 (0.07) | 0.72 (0.22) |
|  | HbR | 0.26 (-0.29) | 0.78 (-0.09) |  |  | 0.28 (-0.27) | 0.31 (-0.25) |  |  | **0.03* (-0.58)** | **0.00* (-0.72)** |  |  | 0.10 (-0.39) | 0.73 (-0.07) |  |  | 0.84 (-0.08) | 0.97 (0.02) |
| 36 | HbO | **0.05* (0.53)** | 0.34 (0.41) |  |  | 0.85 (-0.12) | 0.97 (-0.01) |  |  | **0.00* (0.82)** | **0.00* (0.80)** |  |  | 0.96 (0.02) | 0.09 (0.36) |  |  | 0.84 (0.20) | 0.72 (0.23) |
|  | HbR | 0.17 (-0.37) | 0.46 (-0.31) |  |  | 0.15 (-0.39) | 0.35 (-0.24) |  |  | **0.01* (-0.69)** | **0.00* (-0.78)** |  |  | 0.19 (-0.30) | 0.13 (-0.35) |  |  | 0.45 (-0.25) | 0.97 (0.01) |
|  |  |  |  |  |  |  |  |  |  |  |  |  |  |  |  |  |  |  |  |
| *S2* |  |  |  |  |  |  |  |  |  |  |  |  |  |  |  |  |  |  |  |
| *Left* |  |  |  |  |  |  |  |  |  |  |  |  |  |  |  |  |  |  |  |
| 5 | HbO | **0.04* (0.56)** | 0.50 (0.25) |  |  | 0.98 (0.02) | 0.97 (0.05) |  |  | 0.16 (0.31) | 0.90 (0.03) |  |  | 0.09 (0.45) | **0.03* (0.46)** |  |  | 0.91 (-0.07) | 0.99 (0.00) |
|  | HbR | 0.49 (0.18) | 0.78 (0.09) |  |  | 0.26 (-0.28) | 0.62 (-0.14) |  |  | 0.16 (-0.32) | 0.08 (-0.37) |  |  | 0.24 (-0.25) | 0.09 (-0.39) |  |  | 0.73 (-0.13) | 0.88 (-0.18) |
| 6 | HbO | 0.35 (0.24) | 0.71 (0.12) |  |  | 0.82 (-0.17) | 0.86 (-0.08) |  |  | 0.67 (0.10) | 0.91 (0.02) |  |  | 0.15 (0.35) | **0.01* (0.59)** |  |  | 0.91 (-0.05) | 0.95 (0.05) |
|  | HbR | 0.31 (-0.26) | 0.95 (-0.03) |  |  | 0.17 (-0.35) | 0.08 (-0.47) |  |  | 0.64 (-0.11) | **0.02* (-0.49)** |  |  | 0.11 (-0.36) | 0.62 (-0.11) |  |  | 0.45 (-0.31) | 0.88 (-0.24) |
| 7 | HbO | 0.15 (0.39) | 0.50 (0.26) |  |  | 0.26 (0.48) | 0.97 (0.05) |  |  | 0.54 (0.16) | 0.32 (0.23) |  |  | 0.08 (0.56) | 0.15 (0.32) |  |  | 0.91 (-0.05) | 0.87 (0.14) |
|  | HbR | 0.93 (0.02) | 0.58 (-0.23) |  |  | 0.23 (-0.32) | 0.29 (-0.29) |  |  | 0.18 (-0.30) | **0.01* (-0.58)** |  |  | **0.00* (-0.86)** | 0.10 (-0.39) |  |  | 0.47 (0.25) | 0.88 (0.24) |
| *Right* |  |  |  |  |  |  |  |  |  |  |  |  |  |  |  |  |  |  |  |
| 24 | HbO | 0.08 (0.47) | 0.34 (0.36) |  |  | 0.51 (-0.32) | 0.97 (0.01) |  |  | 0.09 (0.41) | 0.06 (0.42) |  |  | 0.39 (0.20) | 0.16 (0.30) |  |  | 0.91 (0.08) | 0.72 (0.25) |
|  | HbR | 0.17 (-0.38) | 0.78 (0.10) |  |  | 0.46 (-0.19) | 0.79 (-0.06) |  |  | 0.15 (-0.35) | 0.06 (-0.41) |  |  | 0.15 (-0.33) | 0.61 (-0.12) |  |  | 0.45 (-0.29) | 0.97 (-0.01) |
| 26 | HbO | **0.01* (0.71)** | 0.51 (0.22) |  |  | 0.63 (-0.24) | 0.84 (-0.11) |  |  | 0.08 (0.43) | **0.03* (0.51)** |  |  | 0.39 (0.21) | 0.32 (0.21) |  |  | 0.89 (-0.14) | 0.72 (0.20) |
|  | HbR | 0.16 (-0.39) | 0.71 (-0.15) |  |  | 0.82 (0.05) | 0.70 (-0.10) |  |  | 0.13 (-0.38) | **0.01* (-0.53)** |  |  | 0.19 (-0.30) | 0.73 (-0.07) |  |  | 0.45 (-0.27) | 0.88 (0.15) |
| 30 | HbO | **0.01* (0.82)** | 0.34 (0.44) |  |  | 0.98 (-0.02) | 0.97 (-0.03) |  |  | 0.25 (0.26) | 0.09 (0.37) |  |  | 0.35 (0.22) | 0.30 (0.22) |  |  | 0.83 (0.28) | 0.72 (0.26) |
|  | HbR | 0.38 (-0.26) | 0.58 (0.22) |  |  | 0.60 (0.15) | 0.20 (-0.39) |  |  | 0.15 (-0.34) | **0.00* (-0.67)** |  |  | 0.24 (-0.27) | 0.73 (-0.07) |  |  | 0.45 (-0.36) | 0.88 (0.15) |
|  |  |  |  |  |  |  |  |  |  |  |  |  |  |  |  |  |  |  |  |
| *Inferior parietal* | | |  |  |  |  |  |  |  |  |  |  |  |  |  |  |  |  |  |
| *Left* |  |  |  |  |  |  |  |  |  |  |  |  |  |  |  |  |  |  |  |
| 1 | HbO | 0.11 (0.42) | 0.83 (-0.08) |  |  | 0.98 (0.04) | 0.97 (-0.03) |  |  | 0.90 (0.03) | 0.90 (0.03) |  |  | 0.08 (0.49) | **0.02* (0.52)** |  |  | 0.84 (0.18) | 0.72 (0.21) |
|  | HbR | 0.16 (-0.38) | 0.56 (-0.25) |  |  | 0.20 (-0.32) | 0.27 (-0.30) |  |  | 0.82 (0.06) | 0.34 (-0.20) |  |  | 0.28 (-0.23) | 0.38 (-0.19) |  |  | 0.18 (-0.55) | 0.88 (-0.18) |
| 3 | HbO | 0.45 (0.20) | 0.65 (-0.15) |  |  | 0.85 (0.11) | 0.84 (0.15) |  |  | 0.11 (0.35) | 0.39 (0.19) |  |  | **0.04* (0.65)** | **0.00* (0.81)** |  |  | 0.83 (0.29) | 0.95 (-0.03) |
|  | HbR | **0.03* (-0.56)** | **0.02* (-0.72)** |  |  | 0.09 (-0.46) | **0.02* (-0.65)** |  |  | 0.13 (-0.38) | 0.54 (-0.13) |  |  | 0.33 (-0.20) | **0.01* (-0.68)** |  |  | 0.45 (-0.26) | 0.52 (-0.38) |
| 4 | HbO | 0.14 (0.38) | 0.68 (0.14) |  |  | 1.00 (0.00) | 0.84 (-0.10) |  |  | 0.54 (0.15) | 0.70 (0.10) |  |  | 0.15 (0.36) | **0.01* (0.65)** |  |  | 0.91 (-0.07) | 0.95 (0.11) |
|  | HbR | 0.49 (-0.18) | 0.96 (-0.01) |  |  | 0.64 (-0.12) | 0.47 (-0.19) |  |  | 0.60 (-0.14) | 0.25 (-0.24) |  |  | 0.28 (-0.23) | 0.06 (-0.43) |  |  | 0.72 (-0.16) | 0.52 (-0.40) |
| 10 | HbO | **0.02* (0.61)** | 0.50 (0.26) |  |  | 0.63 (0.23) | 0.84 (0.14) |  |  | 0.14 (0.32) | 0.20 (0.28) |  |  | 0.21 (0.30) | **0.01* (0.71)** |  |  | 0.91 (-0.04) | 0.72 (0.22) |
|  | HbR | **0.03* (-0.57)** | 0.52 (-0.27) |  |  | 0.12 (-0.41) | 0.06 (-0.51) |  |  | 0.13 (-0.38) | **0.01* (-0.53)** |  |  | 0.05 (-0.51) | **0.03* (-0.53)** |  |  | 0.24 (-0.49) | 0.97 (-0.10) |
| 12 | HbO | **0.02* (0.61)** | 0.51 (0.22) |  |  | 0.51 (0.31) | 0.53 (0.37) |  |  | 0.05 (0.50) | 0.10 (0.36) |  |  | 0.11 (0.42) | **0.01* (0.72)** |  |  | 0.84 (0.20) | 0.95 (0.04) |
|  | HbR | **0.01* (-0.70)** | 0.09 (-0.50) |  |  | **0.04* (-0.57)** | **0.01* (-0.71)** |  |  | 0.15 (-0.33) | 0.06 (-0.41) |  |  | **0.02* (-0.61)** | 0.08 (-0.40) |  |  | 0.84 (0.07) | 0.97 (-0.04) |
| *Right* |  |  |  |  |  |  |  |  |  |  |  |  |  |  |  |  |  |  |  |
| 25 | HbO | 0.12 (0.40) | 0.50 (0.25) |  |  | 0.93 (0.08) | 0.97 (-0.01) |  |  | 0.09 (0.40) | **0.03* (0.53)** |  |  | 0.87 (-0.05) | 0.22 (0.26) |  |  | 0.84 (0.17) | 0.72 (0.30) |
|  | HbR | 0.45 (-0.20) | 0.82 (-0.07) |  |  | 0.21 (-0.32) | 0.49 (-0.18) |  |  | 0.74 (0.08) | 0.08 (-0.36) |  |  | 0.90 (-0.03) | 0.15 (-0.32) |  |  | 0.45 (-0.34) | 0.97 (-0.11) |
| 28 | HbO | 0.48 (0.17) | 0.93 (0.02) |  |  | 0.85 (-0.13) | 0.72 (-0.21) |  |  | 0.40 (0.20) | **0.00* (0.70)** |  |  | 0.39 (-0.20) | 0.99 (0.01) |  |  | 0.83 (0.34) | 0.65 (0.42) |
|  | HbR | 0.17 (-0.34) | 0.29 (-0.37) |  |  | 0.15 (-0.37) | 0.47 (-0.18) |  |  | 0.56 (0.15) | 0.45 (-0.15) |  |  | 0.90 (0.03) | 0.44 (-0.17) |  |  | 0.45 (-0.32) | 0.97 (-0.13) |
| 29 | HbO | 0.36 (0.23) | 0.76 (0.10) |  |  | 0.85 (0.14) | 0.85 (0.10) |  |  | 0.05 (0.51) | **0.00* (0.71)** |  |  | 0.14 (0.38) | **0.05* (0.43)** |  |  | 0.83 (0.37) | 0.72 (0.20) |
|  | HbR | **0.01* (-0.68)** | **0.05* (-0.62)** |  |  | **0.00* (-0.92)** | **0.02* (-0.60)** |  |  | 0.17 (-0.30) | **0.00* (-0.86)** |  |  | 0.08 (-0.43) | **0.01* (-0.64)** |  |  | 0.45 (-0.28) | 0.97 (0.02) |
| 33 | HbO | 0.07 (0.47) | 0.65 (0.16) |  |  | 0.98 (-0.03) | 0.86 (-0.08) |  |  | **0.02* (0.59)** | **0.04* (0.48)** |  |  | 0.39 (0.19) | 0.06 (0.41) |  |  | 0.84 (0.23) | 0.65 (0.48) |
|  | HbR | **0.05* (-0.50)** | 0.46 (-0.29) |  |  | 0.14 (-0.39) | 0.08 (-0.44) |  |  | 0.13 (-0.37) | **0.00* (-0.63)** |  |  | 0.11 (-0.36) | 0.17 (-0.31) |  |  | 0.45 (-0.39) | 0.97 (-0.07) |
| 35 | HbO | **0.01 (0.73)** | 0.34 (0.36) |  |  | 0.63 (0.23) | 0.53 (0.35) |  |  | **0.02* (0.62)** | **0.01* (0.66)** |  |  | 0.24 (0.28) | **0.01* (0.68)** |  |  | 0.83 (0.27) | 0.72 (0.35) |
|  | HbR | **0.00* (-1.07)** | 0.06 (-0.55) |  |  | **0.04* (-0.61)** | **0.02* (-0.61)** |  |  | **0.03* (-0.55)** | **0.00* (-0.81)** |  |  | **0.00* (-0.92)** | **0.03* (-0.55)** |  |  | 0.45 (-0.26) | 0.88 (-0.23) |
|  |  |  |  |  |  |  |  |  |  |  |  |  |  |  |  |  |  |  |  |
| Significant p-values (FDR-corrected) are in bold and are marked with an asterisk. Hedges’ g coefficients are indicated in parentheses. | | | | | | | | | | | | | | | | | | | |
